# Expression of Lymphoid Enhancer‐Binding Factor 1 in Cancer‐Associated Fibroblasts Mediates Tumor Growth and Transdifferentiation Toward Squamous Cell Carcinoma in Human Breast Cancer

**DOI:** 10.1002/cam4.70627

**Published:** 2025-01-31

**Authors:** Hiroya Okazaki, Yoshihiro Mezawa, Yang Shi, Mizuki Sakimoto, Zixu Wang, Akane Ishizuka, Kazunari Yamashita, Asahi Satoh, Yu Koyama, Yuki Fukumura, Kazunori Kajino, Atsushi Takano, Tomoyuki Yokose, Toshinari Yamashita, Yohei Miyagi, Yataro Daigo, Akira Katakura, Takehiro Yasukawa, Akira Orimo

**Affiliations:** ^1^ Department of Oral Pathobiological Science and Surgery Tokyo Dental College Tokyo Japan; ^2^ Department of Pathology and Oncology Juntendo University Faculty of Medicine Tokyo Japan; ^3^ Department of Molecular Pathogenesis Juntendo University Graduate School of Medicine Tokyo Japan; ^4^ Department of Human Pathology Juntendo University Faculty of Medicine Tokyo Japan; ^5^ Department of Medical Oncology and Cancer Center Shiga University of Medical Science Shiga Japan; ^6^ Center for Advanced Medicine Against Cancer Shiga University of Medical Science Shiga Japan; ^7^ Center for Antibody and Vaccine Therapy Research Hospital, Institute of Medical Science Hospital, the University of Tokyo Tokyo Japan; ^8^ Department of Pathology, Kanagawa Cancer Center Kanagawa Japan; ^9^ Department of Breast Surgery and Oncology, Kanagawa Cancer Center Kanagawa Japan; ^10^ Molecular Pathology and Genetics Division, Kanagawa Cancer Center Research Institute Kanagawa Japan

**Keywords:** breast cancer, cancer biology, cancer‐associated fibroblasts, microenvironment

## Abstract

**Background:**

Cancer‐associated fibroblasts (CAFs) play a significant role in human breast cancer as a major stromal component. While their role in promoting cancer proliferation and malignancy through interaction with cancer cells in the tumor microenvironment is known, the exact mechanisms behind this interaction are not fully understood.

**Results:**

Our study reveals that lymphoid enhancer‐binding factor 1 (LEF1), a central transcription factor for Wnt/β‐catenin signaling, is expressed in experimentally generated tumor‐promoting CAFs (exp‐CAFs) as well as in CAFs from breast cancer patients, particularly those with a poor prognosis. Notably, LEF1‐expressing CAFs are prevalent in the stroma of squamous cell carcinoma (SCC), an aggressive metaplastic breast cancer subtype with a limited understanding of its development. To investigate the functional importance of LEF1 expression in CAFs, we depleted LEF1 in the exp‐CAFs and subcutaneously implanted them along with breast ductal carcinoma MCF10DCIS.com cells into immunodeficient mice. Depleting LEF1 resulted in reduced xenograft tumor growth, accompanied by decreased cancer‐cell proliferation and angiogenesis in the tumors. Additionally, we observed a significant reduction in the expression of SCC markers p40 (ΔNp63) and cytokeratin 5/6 in the xenograft tumors when LEF1 was depleted in the exp‐CAFs. Furthermore, we identified 13 genes, none of which are established downstream genes of the Wnt/β‐catenin pathway, that exhibit expression patterns similar to LFE1 in our cultured fibroblasts.

**Conclusion:**

In summary, our findings suggest that LEF1 expression contributes to the tumor‐promoting abilities of breast CAFs and that LEF1‐expressing CAFs may drive transdifferentiation toward SCC, possibly through a pathway independent of the canonical Wnt/β‐catenin signaling.

## Graphical abstract

This study focuses on cancer‐associated fibroblasts in human breast cancer. Our findings of the involvement of LEF1‐expressing CAFs in the tumor growth promotion and squamous cell carcinogenesis will expand the understanding on the significance of cancer cell–stromal cell interaction in cancer malignancy.

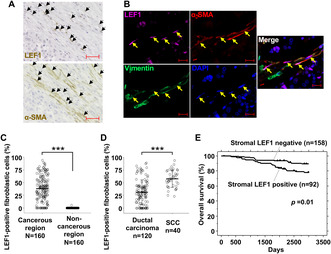

## 1 Introduction

Research on solid tumors have not only focused on the cancer cells but also on the stroma, which evolves alongside cancer cells and plays a role in tumorigenesis. The stroma consists of various nonneoplastic cells, including fibroblasts, immune cells, and endothelial cells [1]. Fibroblasts are abundant in the stroma and provide structural framework. When exposed to cancer cells, fibroblasts undergo changes in their characteristics, distinguishing them from normal fibroblasts [2]. These altered fibroblasts are known as cancer‐associated fibroblasts (CAFs), and numerous studies, including our own [3, 4, 5], have demonstrated their role in promoting cancer malignancy [6]. For instance, our research revealed that CAFs derived from breast carcinomas promote tumor growth and angiogenesis by secreting elevated levels of the C‐X‐C motif chemokine ligand 12 (CXCL12/SDF‐1) [3]. A recent report proposed that fibroblasts influenced by osteopontin produced by breast cancer cells secrete CXCL12, driving cancer cell epithelial‐mesenchymal‐transition and neoangiogenesis [7]. Despite these advancements, our understanding of CAFs remains incomplete, and developing therapeutic strategies targeting CAFs remains a significant challenge. Therefore, gaining a deeper understanding of CAFs is crucial for advancing cancer biology and patient treatment.

Breast cancer, predominantly affecting women, ranks as the most common cancer worldwide [8]. Along cancers such as pancreatic and prostate, breast cancer stands out for its rich stromal composition, with CAFs representing the largest stromal cell population [9]. Notably, metaplastic carcinoma accounts for 0.2%–5% of all breast cancers, and there is no firm treatment for this disease [10]. Squamous cell carcinoma (SCC) of the breast usually develops with rich stroma and is one of the metaplastic carcinoma group tumors of invasive breast carcinomas, alongside low‐grade adenosquamous carcinoma, fibromatosis‐like metaplastic carcinoma, spindle cell carcinoma, and metaplastic carcinoma with heterologous mesenchymal differentiation [11]. Among the metaplastic carcinoma group tumors, SCC is known as a highly aggressive tumor [11]. Generally, it is considered to arise through transdifferentiation from invasive ductal carcinoma [12], although instances of SCC in ductal carcinoma in situ (DCIS) have also been reported [13]. As well as other metaplastic carcinoma group tumors, the vast majority of SCC lacks the expression of estrogen receptor 1 (ER), progesterone receptor (PGR) and erb‐b2 receptor tyrosine kinase 2 (ERBB2), and expresses p63 and p40 (ΔNp63), known as myoepithelial or squamous cell markers, and high‐molecular‐weight cytokeratin, such as cytokeratin 5 and 6 (CK5/6) [14]. In the pathological diagnosis of SCC, immunohistochemical staining of p40 and CK5/6 sometimes assist the correct diagnosis [15, 16], though the majority of SCC cases are histologically typical, such as cancer‐pearl formation and intracellular bridge formation. These markers are also used in diagnosing pulmonary SCC [17], which, such as breast SCC, arises through transdifferentiation from adenocarcinoma [18]. Given the proliferative and challenging‐to‐treat nature of breast SCC, understanding its molecular formation is crucial.

The T‐cell factor/lymphoid enhancer factor (TCF/LEF) family belongs to the high mobility group box (HMG box)‐containing superfamily of transcription factors [19] and serves as a major downstream effector of Wnt/β‐catenin signaling [20]. In mammals, this comprises four proteins: lymphoid enhancer‐binding factor 1 (LEF1), TCF1, TCF3, and TCF4. These proteins form complexes with others and bind to nuclear DNA to regulate the transcription of various genes. A key binding protein of the TCF/LEF family proteins is β‐catenin, which acts as their transcriptional coactivator [19]. LEF1, a central transcription factor in Wnt/β‐catenin signaling, controls the expression of cell cycle and growth‐related genes, such as cyclin D1 and c‐Myc [21]. Interestingly, LEF1 was proposed to function independently of β‐catenin [22], suggesting its complex function. While LEF1 is involved in physiological processes, such as stem cell maintenance, lymphocyte differentiation and proliferation, and organ development, including mammary glands, that requires inductive epithelial–mesenchymal interactions, it is also implicated in cancer development and malignancy [23]. For instances, in poorly differentiated hepatocellular carcinoma, LEF1 is frequently overexpressed and strongly associated with poor prognosis and tumor cell differentiation [24]. Contrary to its canonical role in the Wnt/β‐catenin signaling, LEF1 has been found to directly bind to promoter regions and potentially activate key members of the NOTCH signaling pathway in hepatocellular carcinoma, enhancing self‐renewal capacity, drug resistance, dedifferentiation, and invasion [24]. In gastric cancer, asporin, which is upregulated at various stages of gastric cancer, has been suggested to suppress the cancer cell apoptosis and promote cell proliferation by activating LEF1‐mediated gene transcription independently of β‐catenin [25]. While LEF1 overexpression has been observed in various cancer cells and extensively studied for its altered function [23], its involvement in cancer stroma was only documented in a publication where *Lef1* expression was increased in murine dermal fibroblasts cocultured with human esophageal cancer cells [26]. However, this study did not investigate the tumor growth potential associated with LEF1 expression in fibroblasts.

In our study, we observed a significant presence of LEF1‐positive CAFs within the stroma of human breast cancers, particularly in cases of SCC. Furthermore, through experiments utilizing our experimentally generated CAF (exp‐CAF) line and xenograft models, we demonstrated that LEF1 expression in the exp‐CAFs plays a role in promoting tumor growth and is linked to SCC marker‐positive cancer cells. Our findings suggest that aberrant LEF1 expression in CAFs may drive tumor proliferation and contribute to the transdifferentiation process toward SCC in breast cancer.

## 2 Materials and Methods

### 2.1 Cells

The human exp‐CAFs and counterpart fibroblasts (exp‐CPFs) used in this study were previously described [5]. Fibroblasts and 293 T cells were cultured in DMEM (Nacalai Tesque [Kyoto, Japan], 08459–64) supplemented with 10% fetal bovine serum (FBS) and 1% penicillin–streptomycin (Nacalai Tesque, 26253‐84). Breast ductal carcinoma MCF10DCIS.com (DCIS.com) cells were cultured in DMEM/Ham's F‐12 medium (Nacalai Tesque, 11581‐15) with 5% FBS and 1% penicillin–streptomycin. DCIS.com cells express tdTomato proteins and were utilized in our previous research [5].

### 2.2 Reverse‐Transcription Quantitative PCR

Total RNA was extracted from cultured cells using NucleoSpin RNA (TaKaRa). Subsequently, cDNA was synthesized from the total RNA using PrimeScript RT reagent Kit (TaKaRa) and subjected to reverse‐transcription quantitative PCR (RT‐qPCR) using Fast SYBR Green Master Mix (Thermo Fisher Scientific) to assess the expression levels of target genes. *GAPDH* was utilized as a reference gene to normalize their expression levels. Table S1 provides the PCR primer sequences.

### 2.3 Western Blotting

Total cellular lysates were obtained from cultured cells using RIPA buffer, followed by fractionation via SDS‐PAGE and transfer onto PVDF membranes (Millipore). The membranes were then blocked with BLOCKING ONE solution (Nacalai Tesque, 03953) before being incubated with antibodies to detect target proteins. Table S2 provides the information on antibodies. Protein bands were visualized using Immobilon Classico and Forte Western HRP Substrates (Millipore, WBLUC0500 and WBLUF0500) with a ChemiDoc MP device and Image Lab ver. 4.1 software (Bio‐Rad Laboratories). Adjustments of images were made as needed.

### 2.4 Immunofluorescence Imaging of Cultured Cells

Cells cultured on glass coverslips were fixed with 4% paraformaldehyde in PBS for 10 min. Subsequently, they were permeabilized with 0.1% Triton‐X100 in PBS for 5 min, followed by blocking with 1% normal goat serum and 10% FBS in PBS for 1 h at room temperature. The samples were then incubated with anti‐LEF1 antibodies (Table S2), which were diluted in 0.1% normal goat serum and 1 mg/mL bovine serum albumin in TBST, at 4°C overnight. This was followed by incubation with fluorescently labeled secondary antibodies (Table S2) at room temperature for 1 h. Finally, SlowFade Diamond Antifade Mountant with DAPI (Invitrogen, S36964) was applied, and coverslips were mounted onto glass slides. Immunofluorescent images were captured using an All‐in‐One Fluorescence Microscope BZ‐X800 (KEYENCE). For quantitative evaluation of LEF1‐ and α‐SMA‐positive fibroblasts, cells were stained with anti‐LEF1 and anti‐α‐SMA antibodies (Table S2) simultaneously. Then, images were captured, areas covered by α‐SMA signal were defined, and nuclei positive for DAPI and LEF1 were respectively counted in the α‐SMA‐positive area. The percentage of LEF1‐positive cells in α‐SMA positive cells was calculated.

### 2.5 Immunohistochemistry of the Patient Specimens

Thin sections of FFPE specimens from 20 breast cancer patients, who had not undergone preoperative chemotherapy nor hormonal therapy before surgery, were prepared at 3 μm thickness using a microtome. Immunohistochemical staining was conducted according to previously described methods [5] using antibodies in Table S2 to evaluate the presence of LEF1 and α‐SMA in the stroma. Using a microscope at 40× magnification, eight fields rich in LEF1‐positive fibroblasts were captured in both cancerous and noncancerous regions on each slide from the 20 patients. Fibroblasts were morphologically distinguished from tumor cells, leukocytes, and vascular endothelial cells. The total number of fibroblasts and the number of LEF1‐positive fibroblasts were quantified, and the percentage of LEF1‐positive fibroblasts was calculated for each selected field.

### 2.6 Triple Immunofluorescence Staining of the Patient Specimens

Breast cancer patient specimens for immunofluorescence staining were prepared following the procedure used in the immunohistochemistry experiments, with the exception of the step for endogenous peroxidase inactivation. After incubation with primary and secondary antibodies (Table S2), autofluorescence reduction was achieved by immersing the samples in 0.2% Sudan black B (Merck [Darmstadt, Germany]) in 70% ethanol for 30 min. Immunofluorescence images were captured using an Axioplan 2 microscope (Carl Zeiss [Göttingen, Germany]) and appropriately adjusted using ZEN Pro software (Carl Zeiss).

### 2.7 Tissue Microarray of the Patient Specimens

Tissue microarrays were created using formalin‐fixed tumor specimens from 250 breast cancer patients. After morphologically selecting tissue areas using hematoxylin and eosin‐stained samples, microarray blocks were generated from paraffin embedded tissue cores (diameter 0.6 mm; height 3–4 mm) using a tissue microarrayer (Beecher Instruments). The clinical stage of the breast cancer samples was determined based on the Union for International Cancer Control TNM classification. Thin sections prepared from the blocks were subsequently subjected to immunohistochemical staining for LEF1 (Table S2). The assessment of specimens was conducted by researchers who were unaware of the sample information prior to evaluation. The terms “stromal LEF1‐positive” or “stromal LEF1‐negative” were defined based on a cutoff rate of 10% positive staining of the total stromal area. Additionally, LEF1 staining in cancer cell area was examined with a cutoff rate of 10% positive staining of the total cancer cell area.

### 2.8 Gene Silencing Using shRNA in Cultured Cells

LEF1 expression was suppressed through the transduction of shRNAs using a lentivirus packaging system, following previously described methods [5]. The sequences of the shRNA constructs for LEF1 knockdown are provided in Table S3. The shRNA constructs were inserted into pLKO1.hygro vector (a gift from Bob Weinberg [Addgene_24150]) utilizing *Age*I and *Eco*RI restriction sites. As a negative control, a nonmammalian sequence shRNA (shCtrl) [5] was employed. The efficacy of LEF1 expression knockdown was assessed using RT‐qPCR and western blotting techniques.

### 2.9 Animal Experiments

Six‐week‐old male NOD/Shi‐scid/IL‐2Rγnull (NOG) mice were obtained from CLEA Japan Inc. (Tokyo) and housed under sterile and specific pathogen‐free conditions. To generate xenograft tumors, 9 × 10^4^ DCIS.com cancer cells and 2.7 × 10^5^ fibroblasts mixed in 44% Matrigel (Corning) were subcutaneously implanted into NOG mice. Tumor volume was calculated using the formula: 4/3π(*x*/2)(*y*/2)^2^, where *x* and *y* represent the major and minor axes of the tumor, respectively. Measurement began 1 week after implantation. After 24 days, tumors were excised from the mice, and their wet weights were determined. Subsequently, the tumors were fixed in 10% formalin/100 mM sodium phosphate buffer (pH 7.2) and embedded in paraffin.

### 2.10 Immunohistochemical Staining and Quantitative Analysis of Xenograft Tumors

Immunohistochemistry procedures were conducted as previously outlined [5]. Tumor sections were subjected to antigen activation by incubating in citrate buffer (pH 6.0) at 121°C and 0.2 MPa for 20 min for Ki‐67, CD31, and CK5/6 staining, and in Tris‐EDTA buffer (pH 9.0) at 95°C for 20 min for p40 staining. Primary antibodies for these proteins are listed in Table S2. Quantitative analyses were conducted as described previously [5]. Eight fields abundant in Ki‐67‐, CD31‐, CK5/6‐, or p40‐positive cells were captured and analyzed per xenograft tumor using trained ImageJ software. For Ki‐67 and p40 analyses, cancer cells were identified based on the round shape of the nuclei, and the ratio of positive cancer cells to total cancer cells was determined. For CD31 analysis, microvessel‐occupied areas, including CD31‐positive cells, were measured. For CK5/6 analysis, stromal areas (inter‐cancerous foci) were initially excluded, and then CK5/6‐positive areas within the cancer cell regions (intra‐cancerous foci) were quantified. As the CK5/6 antibody stains the cytoplasm, areas of positive staining were measured instead of counting the positive cell number.

### 2.11 RNA Sequencing and Data Processing

RNA sequencing (RNA‐seq) was conducted as previously described [5]. Adaptor sequences were eliminated using TrimGalore (https://www.bioinformatics.babraham.ac.uk/projects/trim_galore/), and low‐quality reads (*Q* < 20) were trimmed and resulting reads shorter than 20 bases were discarded using FastqPuri [27]. Reads were then aligned to the human genome GRCh38.109 and quantified using HISAT2 [28] and RSEM [29], producing output data comprising expected read counts and transcripts per million (TPM). Subsequently, certain lines in the data were excluded: those containing “pseudogene,” lines with zero expected counts in all samples, and lines lacking hgnc name. The processed output data were then utilized for differential gene expression analysis using DESeq2 [30], retaining genes with read counts equal to and greater than the number of samples. Differentially expressed genes with | Log_2_ fold‐change (FC) | > 1 and adjusted *p*‐value (*p*_adj_) < 0.05 were extracted and depicted through heatmap visualization using R.

### 2.12 Analysis of Published Single‐Cell RNA‐Seq Data

We utilized an integrated pan‐cancer single‐cell information web browser (https://gist‐fgl.github.io/sc‐caf‐atlas/) developed by Luo et al. [31]. Within the browser interface, UMAP was conducted by selecting “fibroblasts only” from the pull‐down menu and “Cell Type (Detailed)” from the annotation menu. Subsequently, the gene symbols of interest were inputted into the “gene symbol” field to generate the UMAP plots for each gene.

## 3 Results

### 3.1 Experimentally Generated CAFs Express LEF1

**FIGURE 1:**
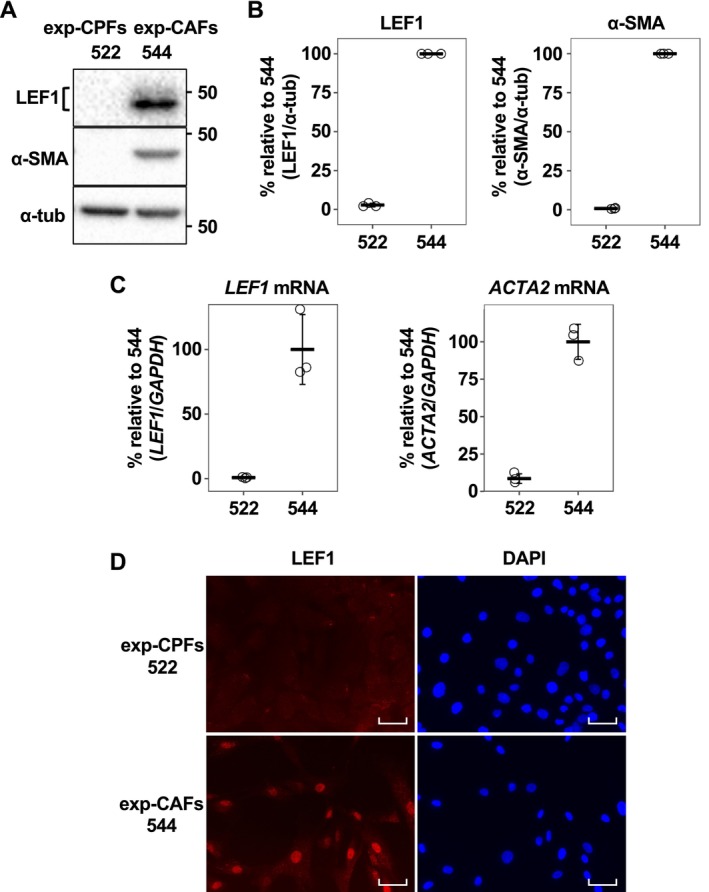
Lymphoid enhancer‐binding factor 1 (LEF1) expression in experimentally generated cancer‐associated fibroblasts (exp‐CAFs). (A) Western blotting analysis to assess LEF1 and α‐smooth muscle actin (α‐SMA) expression in experimentally generated counterpart fibroblast (exp‐CPF) 522 and exp‐CAF 544 cells, with α‐tubulin (α‐tub) as a loading control. (B) Evaluation of western blot bands using three independently harvested sample sets. Band intensities of LEF1 and α‐SMA were normalized to that of α‐tubulin. The normalized intensities of LEF1/α‐tubulin and α‐SMA/α‐tubulin from 544 cells were set as 100 for each set, and values from 522 cells were calculated relative to these. Results are presented as dot plots with the means (thick horizontal lines) and SD (error bars). (C) Reverse‐transcription quantitative PCR analysis to measure *LEF1* and *ACTA2* expression levels. The expression levels of *LEF1* and *ACTA2* mRNAs were normalized against those of *GAPDH* mRNA. The normalized levels of *LEF1*/*GAPDH* and *ACTA2*/*GAPDH* in 544 cells from three independent preparations were averaged and expressed as 100, with values from all samples relative to these. Results are presented similarly to (B). (D) Immunofluorescence imaging of LEF1 in 522 and 544 cells, with nuclei stained with DAPI. Scale bars represent 50 μm.

In this study, we utilized an exp‐CAF line, denoted as exp‐CAF 544 cells [5]. These cells were derived experimentally from immortalized human mammary fibroblasts that underwent prolonged incubation with MCF‐7‐ras human breast cancer cells under the skin of immunodeficient nude mice [4]. Exp‐CAF 544 cells exhibited the capacity to support tumor proliferation [4]. As a counterpart fibroblast line, we employed exp‐CPF 522 cells which underwent subcutaneous incubation as exp‐CAF 544 cells but without the presence of cancer cells. These cells did not show the ability to promote tumor growth [4]. Our previous report indicated that 1936 genes were significantly upregulated (| log_2_FC | > 1, *p*_adj_ < 0.05) in 544 cells compared to 522 cells based on RNA‐seq (Figure S1A) [5]. Notably, *LEF1* emerged among the top 5% of these upregulated genes, exhibiting 7.45‐log_2_FC (544/522) difference in the expression levels, due to substantial upregulation in 544 cells (Figure S1A,B). Conversely, expression levels of *TCF3* and *TCF4* were comparable between 544 and 522 cells (Figure S1B). Although *TCF1* expression seemed induced in 544 cells, it is not as pronounced as *LEF1*. Consequently, among the TCF/LEF family, *LEF1* was prominently induced in the exp‐CAFs. Validation through western blotting and RT‐qPCR analyses confirmed the RNA‐seq findings. LEF1 protein bands were detected in 544 cells, but barely detectable in 522 cells (Figure 1A,B), and the levels of *LEF1* mRNA were mirrored the western blotting observations (Figure 1C). Furthermore, immunofluorescence microscopy indicated that LEF1 was localized within the nuclei of 544 cells (Figure 1D), suggesting that LEF1 in the exp‐CAFs may function as a transcription factor influencing the expression of other genes. Additionally, α‐SMA (gene name: *ACTA2*), commonly utilized as a marker for myofibroblastic CAFs (myCAFs) [2], is expressed in 544 cells (Figure 1A–C), as previously described [5]. About 50% of α‐SMA‐positive 544 cells were LEF1‐positive when they were grown overconfluent (Figure S2). In addition, LEF1 expression was observed in other exp‐CAF lines (Figure S3A–C).

### 3.2 LEF1 Is Expressed in Breast Cancer Patient CAFs, and LEF1‐Positive CAFs Are Abundant in the SCC Subtype

**FIGURE 2:**
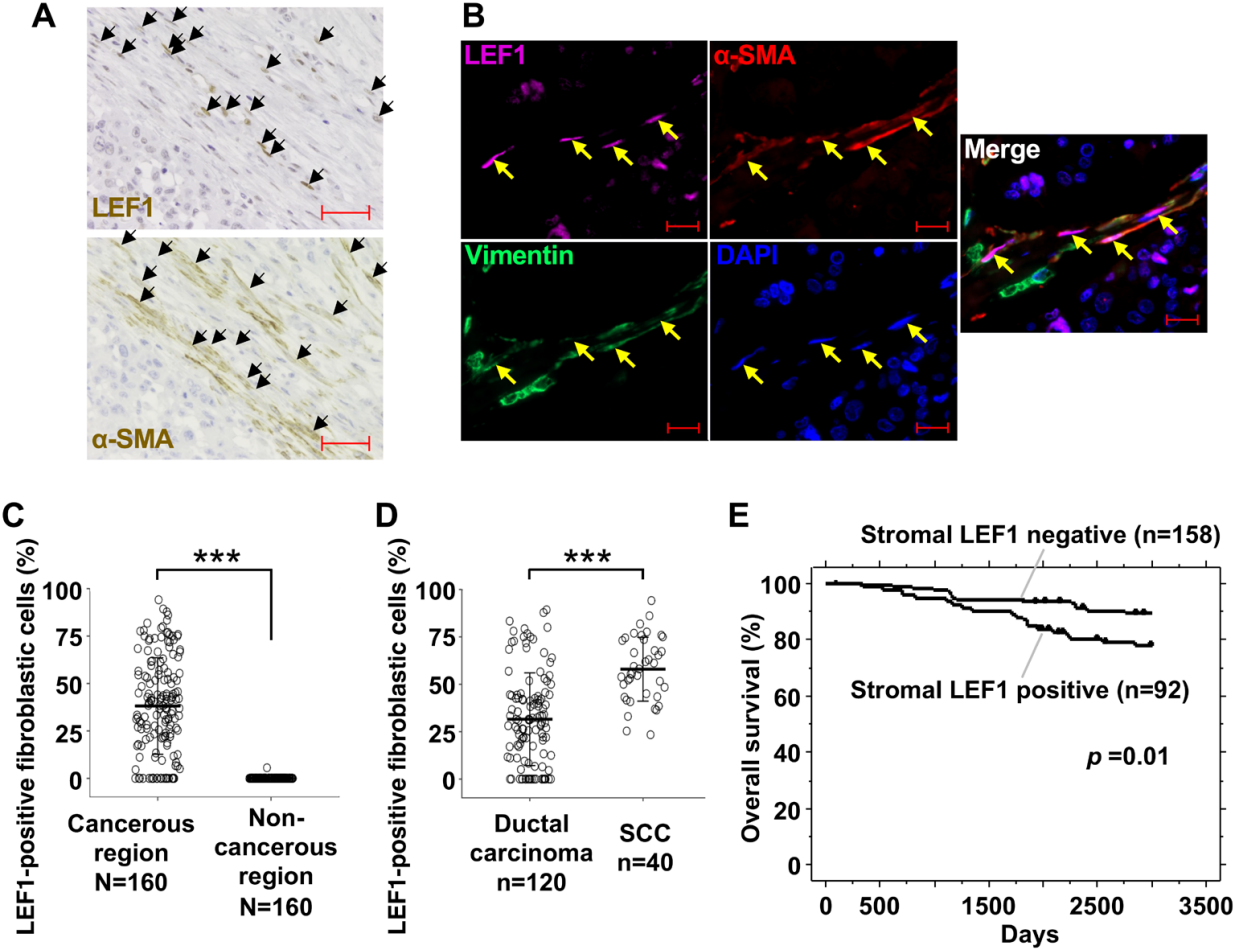
LEF1 expression is upregulated in human breast CAFs. (A) Representative immunohistochemical analysis of breast cancer specimens. Upper and lower panels depict sections of a patient specimen stained with anti‐LEF1 and anti‐α‐SMA antibodies, respectively. LEF1‐ and α‐SMA‐positive cells with a fibroblastic shape are indicated by arrows in the upper and lower panels, respectively. Scale bars represent 50 μm. (B) Multiple fluorescent immunostaining of a breast cancer specimen section with anti‐LEF1, anti‐α‐SMA, and antihuman vimentin antibodies. Nuclei were stained with DAPI. Cells positive for all three antibodies are indicated by arrows. Scale bars represent 20 μm. (C) Quantification of LEF1‐positive cells with a fibroblastic shape in cancerous and noncancerous regions of 20 patients with breast cancer. Breast tissue was immunohistochemically stained for LEF1, and eight fields rich in LEF1‐positive fibroblastic cells were selected from each specimen. The total number (*N*) of analyzed fields was 160 in both cancerous and noncancerous regions. LEF1‐positive and ‐negative fibroblastic cells were counted in the selected fields, and the percentage of positive cells in each field was plotted as dot plots with the means (thick horizontal lines) and SD (error bars). ***p < 0.001 by paired *t*‐test. (D) The percentage of LEF1‐positive fibroblastic cells in the cancerous regions was compared between 15 cases of breast ductal carcinoma and 5 cases of squamous metaplastic carcinoma. The numbers (*n*) of analyzed fields in the former and latter were 120 and 40, respectively. Data are presented similarly to (C). ***p < 0.001 by Welch's *t*‐test. (E) Kaplan–Meier survival plot of 250 human breast cancer patients based on stromal LEF1 staining of their tumors. When ≥ 10% of the stromal region was positive for LEF1 staining, specimens were considered positive (*p* = 0.01 by log‐rank test).

Since we identified LEF1 expression in exp‐CAF 544 cells, we proceeded to investigate the presence of LEF1‐positive CAFs in the stroma of human breast tumor specimens by immunohistochemistry. We observed nuclear LEF1‐positive, elongated spindle‐shaped cells near the areas occupied by cancer cells (Figure 2A). In addition, staining of a section from the same specimen with α‐SMA indicated that the LEF1‐positive cells with fibroblastic morphology that also appeared to be α‐SMA‐positive were present (Figure 2A). These characteristics strongly suggest their identity as LEF1‐expressing CAFs. To support this observation, we performed triple immunofluorescence staining with LEF1, α‐SMA, and a fibroblast maker, vimentin. As shown in Figure 2B, spindle‐shaped cells that were simultaneously positive for the three proteins were detected, confirming the presence of LEF1 (and α‐SMA)‐positive CAFs within breast cancer tumors. In addition, we conducted a reanalysis of single‐cell RNA‐seq data encompassing various cancer types [31] to examine the expression profiles of TCF/LEF family genes in fibroblasts. Our analysis revealed that *LEF1* expression was elevated in CAFs, particularly those categorized as myCAFs and inflammatory CAFs (iCAFs), compared to normal fibroblasts (Figure S4A,B). Conversely, *TCF1*, *TCF3*, and *TCF4* exhibited more uniform pattern in CAFs and normal fibroblasts (Figure S4C–E). These results essentially support our findings in the cultured cell and patient sample analyses (Figures 1 and 2).

Next, we examined the proportion of LEF1‐positive fibroblasts using immunohistochemistry in 20 breast cancer patient samples with α‐SMA‐positive tumor stroma. Among them, there were 5 SCC cases and 15 ductal carcinoma cases, the latter of which included 12 invasive ductal carcinoma cases, 2 DCIS cases, and 1 tubular carcinoma case (Table S4). While LEF1‐positive fibroblasts (cells with a fibroblastic shape) were effectively absent in noncancerous regions (average: 0.03%; Figure 2C), they were abundantly present in the stroma of cancerous regions (average: 38.1%). Moreover, we observed a significantly higher proportion of LEF1‐positive fibroblasts in the cancerous stroma in SCC (average: 57.9%; Figure 2D) compared to ductal carcinoma cases (average: 31.5%). These results suggest an association between LEF1‐positive fibroblasts and breast cancer cells, particularly in SCC.

### 3.3 Stromal LEF1 Expression Correlates With Poor Prognosis in Breast Cancer

To investigate the association between LEF1 expression in the stroma and prognosis in breast cancer, we conducted tissue microarray using tissue specimens from 250 breast cancer patients distinct from the previously mentioned 20 patients (Table S5). Kaplan–Meier survival analysis revealed significantly poorer survival outcomes in the patient group with positive stromal LEF1 staining than those with negative staining (Figure 2E). Consistently, using three prognostic factors that showed statistical significance in the univariate Cox proportional hazards model analysis, the multivariate analysis demonstrated a significant difference between stromal LEF1‐positive and ‐negative cases (Table 1). Because of the limited number of SCC cases among the 250 patients and heterogeneity of adenocarcinoma and SCC within the tumors of the SCC cases, a direct comparison between SCC and ductal carcinoma cases was not feasible. In addition, patient survival and positive LEF1 staining in the cancer cell region was not associated (Figure S5). Taken together, these findings suggest that stromal LEF1 expression is correlated with poor prognosis in breast cancer.

**TABLE 1:** Cox proportional hazards model analysis of prognostic factors in patients with breast cancer.

Variables	Hazards ratio	95% CI	Unfavorable/Favorable	*p*
Univariate analysis				
Stromal LEF1 staining	2.315	1.199–4.47	Positive/Negative	0.0124*
Age (years)	1.37	0.674–2.784	65‐/‐64	0.3845
Luminal type	1.251	0.588–2.66	Negative/Positive	0.5612
HER‐2/neu status	1.131	0.496–2.538	Positive/Negative	0.7695
T‐factor	3.576	1.39–9.201	T2‐3/T1	0.0082*
N‐factor	5.344	2.435–11.728	N1‐2/N0	< 0.0001*
Multivariate analysis				
Stromal LEF1 staining	2.027	1.038–3.957	Positive/Negative	0.0385*
T‐factor	2.138	0.8–5.709	T2‐3/T1	0.1296
N‐factor	4.505	2.016–10.07	N1‐2/N0	0.0002*

* *p* < 0.05.

### 3.4 The Tumor‐Promoting Ability of Exp‐CAF 544 Cells Is Attenuated by LEF1 Suppression

**FIGURE 3:**
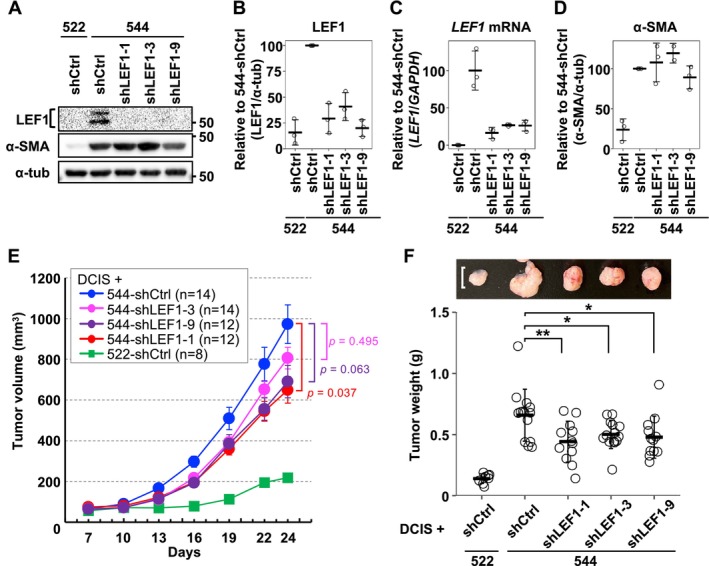
shRNA‐directed LEF1 knockdown attenuates the tumor‐promoting ability of exp‐CAF 544 cells. (A) Western blot results for LEF1 and α‐SMA expression in 544 cells expressing shRNAs targeting LEF1 (shLEF1‐1, ‐3, and ‐9) and 522 and 544 cells expressing control shRNA (shCtrl). α‐Tubulin was used as a loading control. (B) Evaluation of western blot band intensities using three independent experiments. The band intensity of LEF1 was normalized with that of α‐tubulin. The normalized intensity of LEF1/α‐tubulin in 544 cells expressing shCtrl was set as 100, and those in other cells were calculated relative to this at each experiment set. Data from three experiments are presented as dot plots showing the means (thick horizontal lines) with SD (error bars). (C) Reverse‐transcription quantitative PCR analysis of *LEF1* mRNA expression levels from three independent experiments. *LEF1* levels were normalized against *GAPDH*. The normalized levels of *LEF1*/*GAPDH* in 544 cells expressing shCtrl in three independent preparations were averaged and expressed as 100, and those of all samples were calculated relative to them. Data are presented as dot plots showing the means (thick horizontal lines) with SD (error bars). (D) Evaluation of α‐SMA protein levels. Quantitation was performed similarly to (B). (E) Subcutaneous injection of 522 and 544 cells expressing the indicated shRNAs into NOD/Shi‐scid/IL‐2Rγnull (NOG) mice with MCF10DCIS.com (DCIS.com) cells to form xenograft tumors. Tumor volume was measured percutaneously on the indicated days. The number of tumors in each group (*n*) is indicated in the inset. Statistical analysis was performed between the groups with 544 cells using Steel's test. *p* = 0.037 (544‐shCtrl vs. 544‐shLEF1‐1), *p* = 0.495 (544‐shCtrl vs. 544‐shLEF1‐3), and *p* = 0.063 (544‐shCtrl vs. 544‐shLEF1‐9). Error bars represent SEM. (F) Weight of tumors extracted 24 days after implantation. Horizontal thick lines represent the means of tumor weight in each group with SD error bars. **(adjusted *p*_adj_ < 0.01), *(*p*_adj_ < 0.05) by Dunnett's test. Examples of extracted tumors are shown above the graph. The white scale bar represents 10 mm. The numbers of tumors are the same as in (E).

Since LEF1‐positive CAFs were identified in the tumor stroma of breast cancer patients (Figure 2), we aimed to investigate the significance of LEF1 expression in CAFs using our exp‐CAF system. Initially, we examined ten shRNAs targeting LEF1 and identified three shRNAs that efficiently suppressed LEF1 expression without substantially affecting cell growth (shLEF1‐1, ‐3, and ‐9). As shown in Figure 3A–C, LEF1 expression was suppressed at both protein and mRNA levels in exp‐CAF 544 cells expressing each of the three shRNAs. Upon knockdown of LEF1 expression, levels of α‐SMA did not appear to be modified systematically (Figure 3A,D and Figure S6). Furthermore, the mRNA levels of typical iCAF markers, including *CXCL1*, *interleukin 6* (*IL6*), and *leukemia inhibitory factor* (*LIF*) [2], were not systematically changed in LEF1‐suppressed 544 cells (Figure S6). These data suggest that LEF1 is not involved in the expression of these CAF markers in 544 cells.

To examine whether LEF1 expression in exp‐CAF 544 cells correlates with their ability to promote tumor proliferation, we employed a xenograft model using immunodeficient NOG mice. DCIS.com breast cancer cells were combined with the exp‐CAFs with or without LEF1 knockdown and injected subcutaneously. Xenograft tumors formed with 544 cells expressing shLEF1 (544‐shLEF1) exhibited a trend of tumor growth retardation compared to those expressing shCtrl (544‐shCtrl), and the mean tumor volumes with 544‐shLEF1‐1, ‐3, and ‐9 were 67%, 83%, and 71% of the mean volume with 544‐shCtrl, respectively, on day 24 after implantation (Figure 3E). Crucially, upon extracting tumors from mice at the end of the time course, the mean weight of the tumors formed with all three LEF1‐targeting shRNAs was significantly reduced compared to the mean weight of those with shCtrl (67% with shLEF1‐1, 76% with shLEF1‐3, and 72% with shLEF1‐9; Figure 3F). These results indicate that LEF1 overexpression mediates the tumor‐promoting ability of exp‐CAF 544 cells.

**FIGURE 4:**
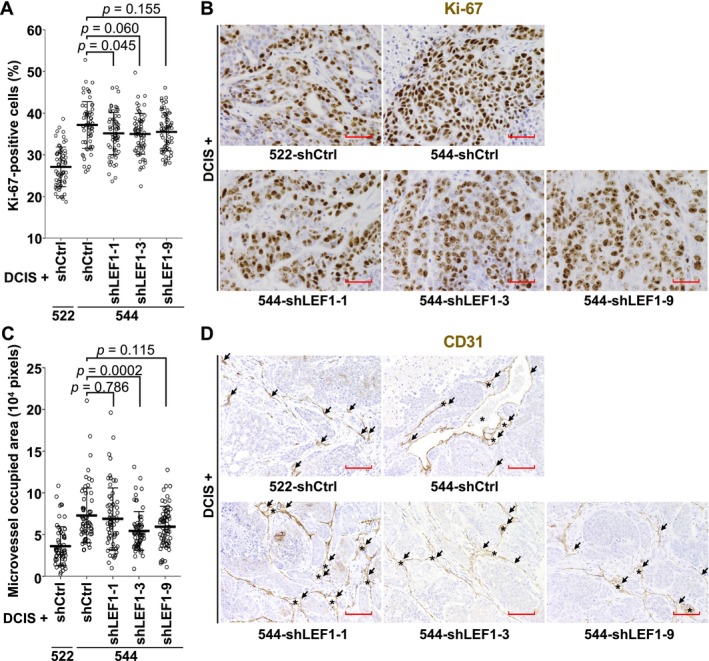
Cancer cell proliferation and neoangiogenesis in xenograft tumors formed with exp‐CAF 544 cells upon LEF1 suppression. (A) Eight fields containing a high density of Ki‐67‐positive cells were selected from tumor sections generated with DCIS.com cells and 522 or 544 cells expressing the indicated shRNAs. After identifying cancer cells based on nuclear shape, the percentage of Ki‐67‐positive cells to Ki‐67‐positive and ‐negative cells was calculated and plotted. The number of observed fields was 64 (the number of tumors: 8) for all groups. Horizontal thick lines represent the means of each group with SD error bars. *p* = 0.045 (544‐shCtrl vs. 544‐shLEF1‐1), *p* = 0.060 (544‐shCtrl vs. 544‐shLEF1‐3), and *p* = 0.155 (544‐shCtrl vs. 544‐shLEF1) by Dunnett's test. (B) Examples of immunohistochemical Ki‐67‐staining images of xenograft tumor sections (brown). Hematoxylin staining was also performed (purple). Scale bars represent 50 μm. (C) Eight fields rich in CD31 staining were selected from each tumor section, and CD31‐positive cell‐surrounded areas, including CD31‐positive cells, were measured as microvessel‐occupied areas. The number of observed fields and data are presented as in (A). *p* = 0.786 (544‐shCtrl vs. 544‐shLEF1‐1), *p* = 0.0002 (544‐shCtrl vs. 544‐shLEF1‐3), and *p* = 0.115 (544‐shCtrl vs. 544‐shLEF1) by Steel's test. (D) Examples of immunohistochemical CD31‐staining images of xenograft tumor sections. Hematoxylin staining (purple) was also performed. Arrows and asterisks indicate CD31‐positive cells (brown) and large microvessels, respectively. Scale bars represent 100 μm.

We further investigated the effect of LEF1 suppression in the exp‐CAFs on cancer cell proliferation and neoangiogenesis. Resected tumors were immunohistochemically stained for the cell proliferation indicator Ki‐67 and the vascular endothelial cell marker CD31. Xenograft tumors formed with 544‐shLEF1‐1 exhibited a significantly lower proportion of Ki‐67‐positive cells compared to those formed with 544‐shCtrl (Figure 4A,B). Tumors generated with 544‐shLEF1‐3 and ‐9 showed trends of reduced cell proliferation without statistical significance. Angiogenesis in xenograft tumors was assessed by measuring CD31‐positive cell‐surrounding areas, including positive cells. As shown in Figure 4C,D, tumors formed with 544‐shLEF1‐3 exhibited significantly decreased microvessel‐occupied areas compared to those formed with 544‐shCtrl. In addition, the occupied areas were smaller in tumors with shLEF1‐1 and ‐9 without statistical significance. Overall, these data are consistent with the idea that aberrant LEF1 expression in 544 cells contributes to xenograft tumor proliferation.

### 3.5 SCC Marker‐Expressing Cancer Cells Decrease in Xenograft Tumors Formed by Coinjection With LEF1‐Suppressed Exp‐CAF 544 Cells

**FIGURE 5:**
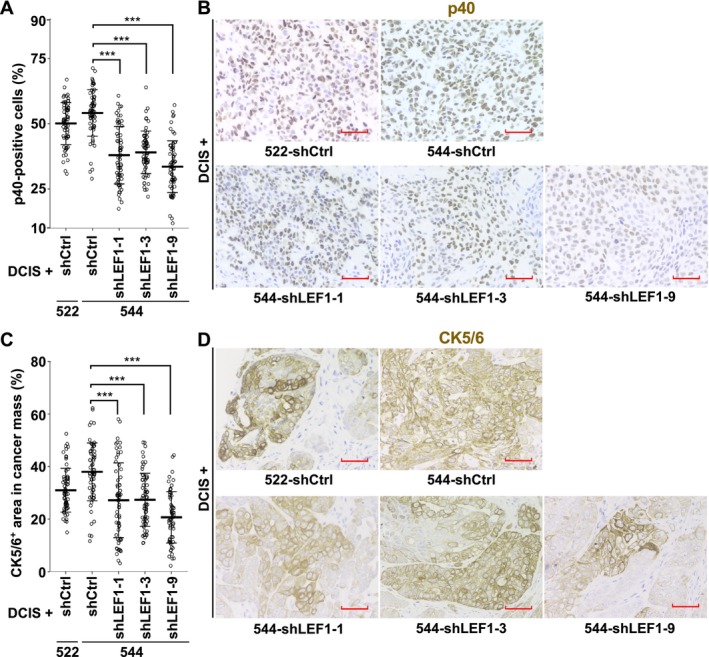
LEF1 knockdown in exp‐CAF 544 cells reduces the number of p40‐and cytokeratin 5/6 (CK5/6)‐positive cancer cells in xenograft tumors. (A) Eight fields with a high density of p40‐positive cells were selected from tumor sections generated with DCIS.com cells and 522 or 544 cells expressing the indicated shRNAs. After identifying cancer cells based on nuclear shape, the percentage of p40‐positive cells to p40‐positive and ‐negative cells was calculated and plotted in the graph. The number of observed fields was 64 (the number of xenografts: 8) for all groups. Horizontal thick lines represent the means of each group with SD error bars. ***(*p*_adj_ < 0.001) by Steel's test. (B) Examples of immunohistochemical staining images of xenograft tumor sections stained with anti‐p40 antibodies (brown). Hematoxylin staining was also performed (purple). Scale bars represent 50 μm. (C) Eight fields containing a high density of CK5/6‐positive cells were selected from tumor sections generated with DCIS.com cells and the indicated fibroblasts. The percentage of CK5/6‐positive (CK5/6^+^) areas within the cancer cell mass was calculated and plotted. The graph presentation follows the same format as in (A). ***(*p*_adj_ < 0.001) by Steel's test. (D) Examples of immunohistochemical staining images of xenograft tumor sections stained with anti‐CK5/6 antibodies (brown). Hematoxylin staining was also performed (purple). Scale bars represent 50 μm.

Given that LEF1‐positive CAFs were more prevalent in SCC compared to ductal carcinoma in breast cancer patients (Figure 2D), we investigated the association between CAF LEF1 expression and breast cancer subtypes using the xenograft tumors described above. Immunohistochemical staining of the tumors with a SCC marker, p40 [15, 17, 32], revealed that while the proportion of p40‐positive cells was 54.1% in the tumors formed with 544‐shCtrl, it decreased to 38.0%, 39.0%, and 33.6% with 544‐shLEF1‐1, ‐3, and ‐9, respectively (Figure 5A,B). Moreover, we assessed them with another SCC marker, CK5/6 [16, 17]. The percentages of CK5/6‐positive areas within the cancer cell areas in tumor sections were 38.0%, 27.2%, 27.3%, and 20.7% for 544‐shCtrl, 544‐shLEF1‐1, 544‐shLEF1‐3, and 544‐shLEF1‐9, respectively (Figure 5C,D). The degree of positivity was notably lower in tumors formed with 544 cells expressing shLEF1 compared to those formed with 544 cells expressing control shRNA in both p40 and CK5/6 staining. These findings indicate that the suppression of LEF1 expression in the exp‐CAFs led to a reduction in the abundance of SCC marker‐positive cancer cells in xenograft tumors.

### 3.6 The Gene Expression Patterns of 13 Genes Are Concordant With LEF1 Expression in Cultured Fibroblasts

**FIGURE 6:**
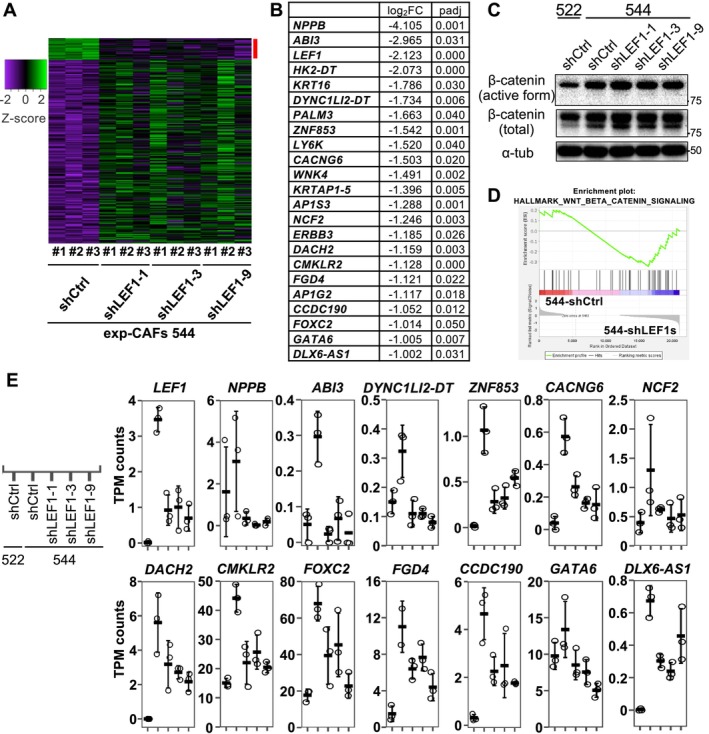
Expression levels of 22 genes were reduced in exp‐CAF 544 cells following LEF1 knockdown. (A) Heatmap of expression levels of differentially expressed genes (| log_2_ FC | > 1; *p*_adj_ < 0.05) between 544‐shCtrl and 544‐shLEF1‐1, ‐3, and ‐9. RNA sequencing (RNA‐seq) results with three independent RNA preparations (#1, #2, and #3) were analyzed. (B) Twenty‐two genes whose expression was significantly downregulated by more than two‐fold upon LEF1 knockdown in 544 cells. Log_2_ FC and *p*_adj_ values are shown. Their positions in the heatmap are indicated by a red line in (A). (C) Gene Set Enrichment Analysis was conducted on 544‐shCtrl and 544‐shLEF1‐1, ‐3, and ‐9 using the gene set for hallmark_Wnt_β‐catenin_signaling. (D) Western blot results for active β‐catenin (unphosphorylated form) and total β‐catenin. Quantitation data are shown in Figure S7. (E) Transcription per million (TPM) counts of *LEF1* and the 13 selected genes in 522‐shCtrl, 544‐shCtrl, 544‐shLEF1‐1, 544‐shLEF1‐3, and 544‐shLEF1‐9. RNA‐seq results from three independent RNA preparations are depicted as dot plots with the means (thick horizontal lines) and SD (error bars). Assignment of lanes in the graphs is indicated next to the *LEF1* graph.

To gain a deeper understanding of how LEF1 depletion in exp‐CAF 544 cells led to the suppression of xenograft tumor, we conducted RNA‐seq analysis to compare the gene expression profiles of 544‐shCtrl and 544‐shLEF1s (shLEF1‐1, ‐3, and ‐9). DESeq2 analysis revealed 217 differentially expressed genes (| log_2_FC | > 1) with statistical significance (*p*_adj_ < 0.05) (Figure 6A). Among them, 22 genes exhibited decreased expression upon LEF1 knockdown (Figure 6B). Notably, none of these genes were targets of the Wnt/β‐catenin pathway. Consistently, the active form of β‐catenin was not downregulated by LEF1 knockdown in 544 cells (Figure 6C and Figure S7), and TPM counts of Wnt/β‐catenin pathway target genes did not consistently decrease with the three shLEF1s except for *FOSL1* (Figure S8). *FOSL1* expression levels decreased in LEF1‐suppressed exp‐CAF 544 cells yet were higher in exp‐CPF 522 cells lacking LEF1 expression compared to exp‐CAF 544 cells (Figure S8), suggesting unlikeliness of the link between LEF1 and *FOSL1* expression. Furthermore, Gene Set Enrichment Analysis (GSEA) [33, 34] did not suggest an association between LEF1 knockdown and the hallmark_Wnt_beta_catenin_signaling gene set (NOM *p‐val*, 0.637; FDR *q‐va*, 0.637; Figure 6D). However, among the 22 downregulated genes, the expression levels of 13 genes, *NPPB1*, *ABI3*, *DYNC1LI2‐DT*, *ZNF853*, *CACNG6*, *NCF2*, *DACH2*, *CMKLR2*, *FOXC2*, *FGD4*, *CCDC190*, *GATA6*, and *DLX6‐AS1*, were lower in 522‐shCtrl than in 544‐shCtrl (Figure 6E). This suggests that the overall expression patterns of these 13 genes in exp‐CAF 544 and exp‐CPF 522 cells correlate with the LEF1 expression patterns. We additionally examined LEF1 binding signature to these genes and around their 5′‐ends in published human LEF1 ChIP‐seq data using ChIP‐Atlas [35]. Binding signatures appear to present at the 5′‐end regions of *NBBP*, *DYNC1LI2‐DT*, *CMKLR2*, and *CCDC190* genes and an intron region of *GATA6* gene (Figure S9). Altogether, these results suggest that LEF1 regulates genes unrelated to the canonical Wnt/β‐catenin signaling in exp‐CAF 544 cells and plays a role in mediating their tumor‐promoting ability.

## 4 Discussion

In this study, we observed expression of LEF1 in exp‐CAF 544 cells derived from human mammary fibroblasts coimplanted with human breast cancer cells in a mouse model [4]. Subsequently, we analyzed tumor specimens from breast cancer patients and found that LEF1‐positive CAFs are abundant and cancerous region‐specific. Notably, LEF1‐positive CAFs were more prevalent in SCC compared to ductal carcinoma. Furthermore, tissue microarray analysis indicated that stromal LEF1 staining serves as an independent prognostic factor for poor outcomes in breast cancer. These findings suggest that LEF1‐expressing CAFs play a role in tumor progression and contribute to SCC formation. Moreover, our observation in the exp‐CAF system and patient specimens align with data in published datasets: through reanalysis of single‐cell RNA‐seq data from various cancers [31], we found that *LEF1* exhibits greater specificity to myCAFs and iCAFs than to normal fibroblasts and that this specificity is clearer to *LEF1* compared to other members of the TCF/LEF family.

Having identified the connection between LEF1 expression and CAFs, we investigated the impact of LEF1 on CAFs by suppressing its expression in exp‐CAF 544 cells. These cells, transduced with either control shRNA or one of three shRNAs targeting LEF1, were coinjected with breast MCF10DCIS.com cells into NOG mice. Notably, xenograft tumors resulting from this experiment exhibited significantly reduced weight and a trend of decreased volume when LEF1 was knocked down. Furthermore, we observed a tendency for diminished proliferative capacity of cancer cells and reduced angiogenesis in tumors with LEF1‐depleted 544 cells. These results indicate that LEF1 expression in the exp‐CAFs promotes tumor growth, potentially by stimulating cancer cell proliferation and blood vessel formation. Given the prevalence of LEF1‐positive CAFs in SCC in breast cancer patient specimens, we evaluated xenograft tumors using SCC markers, p40 and CK5/6. Interestingly, we found significantly lower expression of these proteins in DCIS.com cancer cells coimplanted with exp‐CAF 544 cells with LEF1 knockdown compared to those without knockdown. p40 (ΔNp63), an oncogene and an isoform of p63 with transcriptionally inactive ΔN domain [32], is overexpressed in SCC of various origins, including head and neck, lung, skin, and esophagus [36] and has been associated with breast cancer cell migration [37]. Our observation of a reduction in the number of p40‐positive cancer cells upon LEF1 suppression in exp‐CAF 544 cells suggests that LEF1 expression in the exp‐CAFs may contribute to SCC characteristics in DCIS.com cells coimplanted with them in our xenograft system. In summary, our study reveals the abundance of LEF1‐expressing CAFs in breast cancer stroma and proposes that LEF1 expression plays a role in the tumor‐promoting ability of breast CAFs and potentially mediating transdifferentiation toward SCC.

Previous studies have highlighted CAF involvement in Wnt/β‐catenin signaling, including the activation of signaling in cancer cells via Wnt ligands produced by CAFs [38, 39, 40, 41] and the reliance of CAF function on canonical Wnt/β‐catenin signaling [42, 43]. However, the role of LEF1, a central transcription factor in Wnt/β‐catenin signaling, in CAFs has not been specifically investigated, except in one study [26]. In this study, mouse dermal fibroblasts cocultured with human esophageal cancer cells exhibited upregulated mRNA expression of hyaluronan synthase 2 (*Has2*) and increased secretion of hyaluronan which is a component of the extracellular matrix and contributes to cancer malignancy. Interestingly, this induction of *Has2* was abrogated by knockdown of *Lef1* whose expression was upregulated in the fibroblasts upon coculture [26]. In the current study involving 544 cells derived from human mammary fibroblasts cocultured with human breast cancer cells in a nude mouse model [4], although LEF1 was expressed, knockdown of LEF1 did not significantly alter *HAS2* expression (log_2_FC [544‐shLEF1/544‐shCtrl] = −0.53; *p*_adj_ = 0.39 in DESeq2 analysis). The disparity in *HAS2* expression between the current and previous [26] studies is unclear. This may be due to the differences in coculture method (in vivo and in vitro), tumor cell type (breast and esophagus), and/or origin of fibroblasts (human breast and mouse skin).

To assess the influence of LEF1 expression on CAF characteristics, we compared RNA expression profiles between 544‐shCtrl and 544‐shLEF1 cells. We identified 217 significantly differentially expressed genes, including 22 genes downregulated upon LEF1 knockdown. Notably, none of the 22 genes were known downstream targets of the Wnt/β‐catenin pathway. Consistently, no significant gene enrichment was observed in the Wnt/β‐catenin signaling hallmark between 544‐shCtrl and 544‐shLEF1 using GSEA. Moreover, suppressing LEF1 expression did not reduce the levels of the activate form of β‐catenin in 544 cells. Overall, these findings suggest that LEF1 likely does not function in the Wnt/β‐catenin pathway in the exp‐CAFs. Our previous study on exp‐CAF544 cells suggested that upregulation of CXCL12 and TGF‐β in the fibroblasts supports the tumor‐promoting property in an autocrine manner [4]. Also, breast CAF‐secreted CXCL12 was shown to promote the growth of xenograft tumors formed by patient‐derived CAFs and cancer cells [3] and osteopontin‐activated fibroblast‐derived CXCL12 was proposed to play a role in inducing epithelial‐mesenchymal‐transition and angiogenesis using in vitro assays [7]. In the current study, however, LEF1 depletion in 544 cells did not decrease mRNA expression of CXCL12 and TGF‐β, suggesting they are not involved in LEF1‐medicated tumor‐promoting effect of 544 cells. By contrast, we observed that 13 out of the above 22 genes showed higher expression in exp‐CAF 544 cells compared to exp‐CPF 522 cells. We hypothesize that these 13 genes, which exhibit low expression in 522 cells (no LEF1 expression), high expression in 544 cells (LEF1 overexpression), and decreased expression in 544 cells upon LEF1 knockdown, may influence tumor growth and transdifferentiation into SCC, as they seem to be regulated by LEF1 in our experimental system. Notably, the most significantly downregulated gene upon LEF1 suppression was *NPPB*, encoding B‐type natriuretic peptide (BNP), a well‐known secreted protein acting as a cardiac hormone [44, 45]. Analysis of published ChIP‐seq data using ChIP‐Atlas [35] suggested that LEF1 can bind to ~1.3 kb upstream of this gene. BNP interacts with natriuretic peptide receptor A and when this receptor is bound by BNP, it exerts many effects, such as vascular relaxation [45], angiogenesis [46], and antifibrosis [45, 47]. It was shown that cardiac fibroblasts produce and secrete BNP, which, in turn, inhibits collagen synthesis and fibroblast proliferation [48, 49]. Intriguingly, it has been reported that the *NPPB* expression levels are higher in pancreatic ductal adenocarcinoma‐associated fibroblasts compared to control fibroblasts [50]. Upon treatment with BNP, the former exhibited less pronounced decrease in *ACTA2* expression compared to the latter, suggesting a greater resistance to the antifibrotic effects of BNP in the former cells [50]. Moreover, it has been proposed that a gene product of *NPPB* expressed by CAFs could serve as a potential biomarker in epithelial ovarian cancer [51]. Considering these previous studies alongside our current work, it is possible that BNP secretion from LEF1‐expressing exp‐CAFs suppresses the formation of rigid extracellular matrix and promotes angiogenesis in xenograft tumors, with cancer cells likely favoring such alternations. In addition, it appears there is a lack of reports describing the remaining 12 genes in the stroma. Given that the mRNA expression patterns of these 13 genes align with LEF1 expression levels in our cultured fibroblast system, we infer that they (or some of them) may directly contribute to the tumor‐promoting abilities of exp‐CAF 544 cells and regulate p40 and CK5/6 expression in cancer cells in xenograft tumors.

This study has identified LEF1‐expressing CAFs within the breast cancer stroma and proposed their involvement in tumor growth and SCC formation. A limitation of this study is the lack of a detailed molecular mechanism explaining how these roles are carried out by LEF‐expressing CAFs. Additionally, it also remains unclear how LEF1 expression is induced and sustained in CAFs. It is possible that cancer cell‐derived factors Wnt7a and osteopontin, which were proposed to mediate fibroblast activation to CAFs [7, 52], are involved in these processes. Although the interaction between cells in the tumor microenvironment is complex, we propose that LEF1‐expressing CAFs contribute to the proliferative potential of cancer cells and their ability to undergo transdifferentiation into SCC. Further investigations are needed to elucidate the relationship between aberrantly expressed LEF1 and the genes that exhibit expression patterns similar to LEF1. Such understanding may pave the way for developing therapeutic strategies targeting CAFs and unraveling the mechanisms underlying SCC development.

## Author Contributions

**Hiroya Okazaki:** conceptualization (supporting), data curation (lead), formal analysis (lead), investigation (lead), methodology (lead), validation (lead), visualization (lead), writing – original draft (equal). **Yoshihiro Mezawa:** investigation (supporting), methodology (supporting). **Yang Shi:** formal analysis (supporting), investigation (supporting). **Mizuki Sakimoto:** formal analysis (supporting), investigation (supporting). **Zixu Wang:** methodology (supporting), software (equal). **Akane Ishizuka:** investigation (supporting), methodology (supporting). **Yu Koyama:** funding acquisition (equal), investigation (supporting). **Yuki Fukumura:** investigation (supporting), writing – review and editing (supporting). **Kazunori Kajino:** investigation (supporting). **Atsushi Takano:** investigation (supporting), resources (supporting). **Tomoyuki Yokose:** resources (supporting). **Toshinari Yamashita:** resources (supporting). **Yohei Miyagi:** resources (supporting). **Yataro Daigo:** formal analysis (equal), investigation (supporting), methodology (equal), resources (lead), writing – review and editing (supporting). **Akira Katakura:** funding acquisition (supporting), supervision (supporting). **Takehiro Yasukawa:** conceptualization (equal), data curation (equal), formal analysis (equal), funding acquisition (lead), investigation (supporting), methodology (supporting), project administration (lead), resources (supporting), software (equal), supervision (lead), writing – original draft (lead). **Akira Orimo:** conceptualization (equal), funding acquisition (supporting), supervision (supporting), writing – review and editing (supporting). **Kazunari Yamashita:** investigation (equal). **Asahi Satoh:** investigation (equal).

## Ethics Statement

Use of FFPE tissue specimens for immunohistochemical and immunofluorescence analysis was approved by the Juntendo University ethics review board. For tissue microarray analysis, approval for this study, including the use of clinical materials, was obtained from individual institutional ethics committees. Additionally, the project aimed at establishing tumor tissue microarrays from archival FFPE and surgically obtained tissues, and utilizing them for future research, was approved by the Kanagawa Cancer Center Ethics Committee. The patients included in the breast cancer tissue microarrays underwent surgery at Kanagawa Cancer Center Hospital. Written informed consent was obtained from the patients for the use of their clinical information and for any remaining specimens from clinical examinations, such as archival FFPE specimens. Experiments were carried out in accordance with all guidelines and regulations indicated by the committees and conformed to the provision of the Declaration of Helsinki. Registry and the Registration no. of the study/trial: N/A. Animal experiments were approved by the Animal Research Ethics Committee of the Juntendo University, Faculty of Medicine.

## Conflicts of Interest

T. Yamashita receives honoraria from Chugai Pharmaceutical, Eisai, AstraZeneca, Kyowa Hakko Kirin, Pfizer, Taiho Pharmaceutical, Eli Lilly, Daiichi Sankyo, MSD, Nippon Kayaku, and Novaritis Pharma and research funding through his institution from Chugai Pharmaceutical, AstraZeneca, Kyowa Hakko Kirin, Pfizer, Taiho Pharmaceutical, Eli Lilly, Daiichi Sankyo, Nihonkayaku, Segen, MSD, Ono, Gilead Sciences, and Eisai. T. Yasukawa receives research funding through his institution from Renee Medical Corporation.

## Reproduction of Material

The volcano plot shown in Figure S1 is a reuse of Figure 1 in our previous publication (Koyama et al. [5]). The purpose of presenting the data is to facilitate readers' understanding. Cancer Medicine permits the reuse of the published material provided that full attribution is included in the new work. It is explicitly stated on the website (https://onlinelibrary.wiley.com/page/journal/20457634/homepage/permissions.html). Following the instruction, we have cited the paper in the figure legend of Figure S1A.

## Supporting information


Data S1.

Data S2.

## Data Availability

RNA‐seq raw data will be available in a public repository upon acceptance for publication. RNA‐seq raw data are deposited in GSE287907.
