# Supplementary material for: Expression of Lymphoid Enhancer‐Binding Factor 1 in Cancer‐Associated Fibroblasts Mediates Tumor Growth and Transdifferentiation Toward Squamous Cell Carcinoma in Human Breast Cancer
Source: Cancer Med. 2025 Jan 31;14(3):e70627. doi: 10.1002/cam4.70627 (PMC11783236; doi:10.1002/cam4.70627)
Supplement: Supplementary file 1 — Data S1. [file CAM4-14-e70627-s002.docx]

*** Supplementary Data ***

**Expression of lymphoid enhancer-binding factor 1 in cancer-associated fibroblasts mediates tumor growth and transdifferentiation toward squamous cell carcinoma in human breast cancer**

This document contains:

- Supplementary Tables 1−5 (Tables S1−S5)
- Supplementary Figures 1−9 (Figures S1−S9)

**Table S1. Primers for RT-qPCR**

| Target RNA | | Sequence(5`-to-3`) |
| --- | --- | --- |
| *LEF1* | Forward | CCGAAGAGGAAGGCGATTTAGC |
|  | Reverse | GGTCCCTTGTTGTAGAGGCC |
| *ACTA2* | Forward | GTGTGTGACAATGGCTCTGG |
|  | Reverse | TGGTGATGATGCCATGTTCT |
| *GAPDH* | Forward | TCAACTACATGGTTTACATGTTC |
|  | Reverse | GATCTCGCTCCTGGAAGAT |

**Table S2. Antibodies**

| **Primary antibodies** | | | | | |
| --- | --- | --- | --- | --- | --- |
| Target protein | Product details | | Clone name  (if applicable) | | Experiments |
| LEF1 | Cell Signaling Technology, #2230 | | C12A5 | | Western blotting  Immunofluorescence (cultured cells) |
| LEF1 | Abcam, ab137872 | | EPR2029Y | | Immunohistochemistry (patient specimens)  Immunofluorescence (patient specimens)  Tissue microarray |
| α-SMA | Dako, M0851 | | 1A4 | | Western blotting  Immunofluorescence (cultured cells)  Immunohistochemistry (patient specimens) |
| α-SMA | Invitrogen, PA5-18292 | |  | | Immunofluorescence (patient specimens) |
| α-Tubulin | Sigma, T5168 | | B512 | | Western blotting |
| Vimentin | Dako, IR630 | | V9 | | Immunofluorescence (patient specimens) |
| Active-β-catenin | Millipore, 05-665 | | 8E7 | | Western blotting |
| β-Catenin | Upstate, 05-475 | | 7D8 | | Western blotting |
| Ki-67 | Dako, M7240 | | MIB-1 | | Immunohistochemistry (xenograft tumors) |
| CD31 | Abcam, ab28364 | |  | | Immunohistochemistry (xenograft tumors) |
| CK5/6 | DAKO, M723729-2 | | D5/16 B4 | | Immunohistochemistry (xenograft tumors) |
| p40 | BIOCARE MEDICAL, ACR3066A | | BC28 | | Immunohistochemistry (xenograft tumors) |
| **Secondary antibodies** | | | | | |
| Antibody name | | Product details | | Experiments | |
| anti-rabbit IgG, horseradish peroxidase-linked antibody | | Cell Signaling Technology, #7074 | | Western blotting | |
| anti-mouse IgG, horseradish peroxidase-linked antibody | | Cell Signaling Technology, #7076 | | Western blotting | |
| Alexa Fluor 568-labeled goat anti-rabbit IgG antibody | | Invitrogen, A11036 | | Immunofluorescence (cultured cells) | |
| Alexa Fluor 488-labeled donkey anti-mouse IgG antibody | | Invitrogen A21202 | | Immunofluorescence (patient specimens) | |
| Alexa Fluor 568-labeled donkey anti-goat IgG antibody | | Invitrogen A11057 | | Immunofluorescence (patient specimens) | |
| Alexa Fluor 647-labeled donkey anti-rabbit IgG antibody | | Invitrogen A31573 | | Immunofluorescence (patient specimens) | |
| Alexa Fluor 647-labeled goat anti-mouse IgG antibody | | Invitrogen A21235 | | Immunofluorescence (cultured cells) | |

**Table S3. Oligonucleotides for generating shRNA expression constructs**

| shRNA name |  | Sequence (5'- to -3') |
| --- | --- | --- |
| shLEF1-1 | Sense | CCGGTCGTTGCTGAGTGTACTCTAAACTCGAGTTTAGAGTACACTCAGCAACGTTTTTG |
|  | Antisense | AATTCAAAAACGTTGCTGAGTGTACTCTAAACTCGAGTTTAGAGTACACTCAGCAACGA |
| shLEF1-3 | Sense | CCGGTGCATCCCTCATCCAGCTATTGCTCGAGCAATAGCTGGATGAGGGATGCTTTTTG |
|  | Antisense | AATTCAAAAAGCATCCCTCATCCAGCTATTGCTCGAGCAATAGCTGGATGAGGGATGCA |
| shLEF1-9 | Sense | CCGGGCTGGTCTGCAAGAGACAATTTACTAGTAATTGTCTCTTGCAGACCAGCTTTTTG |
|  | Antisense | AATTCAAAAAGCTGGTCTGCAAGAGACAATTACTAGTAAATTGTCTCTTGCAGACCAGC |

Target sequences in sense oligonucleotides are underlined.

**Table S4. Histopathological information of 20 breast cancer patients**

| No. | Age | Diagnosis | Grade | pT factor | pN factor | ER | PR | HER2 |
| --- | --- | --- | --- | --- | --- | --- | --- | --- |
| 1 | 70 | IDC | NG3, HG3 | 1b | 0 | - | - | - |
| 2 | 56 | IDC | NG2, HG1 | 2 | 0 | - | - | - |
| 3 | 71 | IDC | NG2, HG1 | 1c | 1a | - | - | - |
| 4 | 67 | DCIS | NG2 | is | 0 | - | - | - |
| 5 | 72 | IDC | NG3, HG3 | 2 | 0 | - | - | - |
| 6 | 72 | SCC | - | 4b | 1 or 2a | - | + | - |
| 7 | 61 | SCC | - | 3 | 0 | - | - | - |
| 8 | 65 | SCC | - | 2 | 0 | - | - | - |
| 9 | 51 | SCC | - | 2 | 1a | - | - | + |
| 10 | 69 | SCC | - | 2 | 0 | + | + | - |
| 11 | 44 | IDC | NG1, HG1 | 1c | 0 | + | + | - |
| 12 | 53 | IDC | NG2, HG1 | 1c | 0 | + | + | - |
| 13 | 44 | IDC | NG2, HG2 | 1c | 0 | + | + | - |
| 14 | 84 | IDC | NG1, HG1 | 1a | 0 | + | + | - |
| 15 | 46 | TC | - | 1b | 0 | + | + | - |
| 16 | 57 | IDC | NG3, HG3 | 1 | 0 | - | - | + |
| 17 | 40 | IDC | NG3 | 1mic | 0 | + | + | + |
| 18 | 58 | DCIS | NG2 | is | 0 | + | + | + |
| 19 | 60 | IDC | NG3, HG3 | 1mic | 0 | - | - | + |
| 20 | 41 | IDC | NG3 | 1mic | 0 | - | - | + |

Abbreviations: IDC, invasive ductal carcinoma; TC, tubular carcinoma; SCC, squamous cell carcinoma; DCIS, ductal carcinoma in situ; HG, histological grade; NG, nuclear grade; ER, estrogen receptor; PR, progesterone receptor; HER2, human epithelial growth factor receptor type 2.

**Table S5. Association between stromal LEF1-positivity in breast cancer tissues and patients’ characteristics**

| Parameters |  |  | Stromal LEF1 | | P-value |
| --- | --- | --- | --- | --- | --- |
|  |  | Total | Positive expression | Negative expression |  |
|  |  | n = 250 | n = 92 | n = 158 |  |
| Age (years) | –64 | 187 | 72 | 115 | 0.3677 |
|  | 65– | 63 | 20 | 43 |  |
| Grading | 0 | 41 | 13 | 28 | 0.116 (※) |
|  | 1 | 82 | 26 | 56 |  |
|  | 2 | 65 | 29 | 36 |  |
|  | 3 | 62 | 24 | 38 |  |
| pT factor | T1 | 88 | 25 | 63 | 0.0543 |
|  | T2-3 | 162 | 67 | 95 |  |
| pN factor | N0 | 145 | 52 | 93 | 0.7907 |
|  | N1-2 | 105 | 40 | 65 |  |
| Luminal | Positive | 176 | 66 | 110 | 0.7749 |
|  | Negative | 74 | 26 | 48 |  |
| Her2 status | Positive | 45 | 19 | 26 | 0.4002 |
|  | Negative | 205 | 73 | 132 |  |

(※) Grading 0 and 1 vs. 2 and 3
