# Supplementary material for: Expression of Lymphoid Enhancer‐Binding Factor 1 in Cancer‐Associated Fibroblasts Mediates Tumor Growth and Transdifferentiation Toward Squamous Cell Carcinoma in Human Breast Cancer
Source: Cancer Med. 2025 Jan 31;14(3):e70627. doi: 10.1002/cam4.70627 (PMC11783236; doi:10.1002/cam4.70627)
Supplement: Supplementary file 2 — Data S2. [file CAM4-14-e70627-s001.pdf]

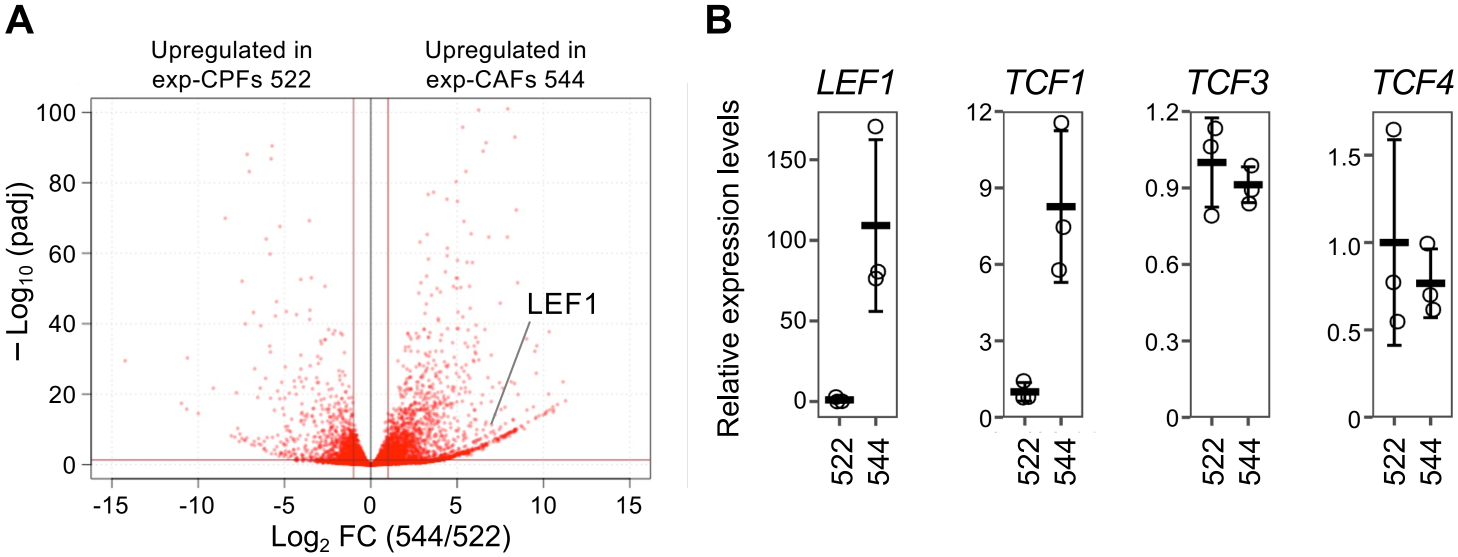

gene	log2FC	padj	gene	log2FC	padj
LEF1	**7.45**	**1.07E-10**	*TCF3*	-0.40	0.01
TCF1	3.38	1.72E-14	*TCF4*	-0.76	0.19

Figure S1

Figure S1. Analysis of gene expression levels in three independent preparations of exp-CPF 522 and exp-CAF 544 cells

(A) Volcano plots showing the differences in the global RNA expression between 522 and 544 cells. RNA-seq results with three independent preparations were examined by DESeq2. X- and Y-axes respectively show the $\log_2$ FC in 544 cells relative to 522 cells and $-\log_{10}$ of the adjusted *p*-value (padj). Log_2 FC of ± 1 (X-axis) and padj of 0.05 (Y-axis) are indicated by brown lines. The dot for LEF1 is indicated. The table shows the values of Log_2 FC and padj of the indicated genes. The volcano plots are the reproduction of Figure 1 in our previous publication (Koyama Y, Okazaki H, Shi Y, Mezawa Y, Wang Z, Sakimoto M, *et al.* Increased RUNX3 expression mediates tumor-promoting ability of human breast cancer-associated fibroblasts. *Cancer Medicine* **2023**;12:18062-77).

(B) Examination of TPM counts of the indicated genes obtained from RNA-seq results (GSE240453) (1). The means of indicated gene expression levels in 522 cells are set as 1, and the relative levels of expression in each sample are shown as dot plots with the means (thick horizontal lines) and SD (error bars).

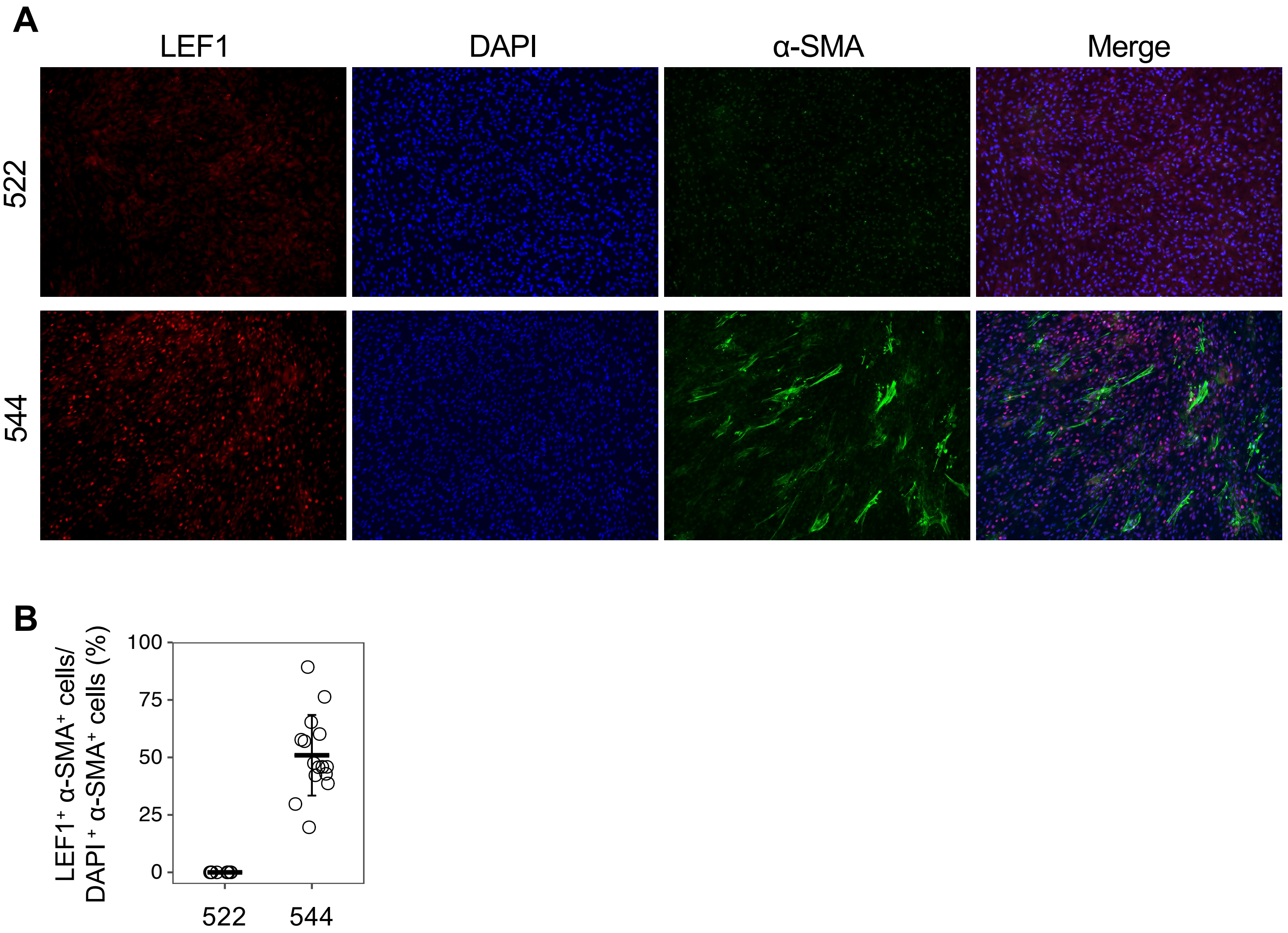

Figure S2

Figure S2. Immunofluorescence imaging analysis of LEF1 and α-SMA in cultured fibroblasts

(A) After cells were confluent, they were incubated for 4 more days, followed by immunofluorescence staining with anti-LEF1 and anti-α-SMA antibodies, with nuclei stained with DAPI. This experiment was performed three times independently. LEF1 expression levels were examined by RT-qPCR at each experiment using RNA samples that were prepared from cells 2 days after they were confluent. Three and five areas for 522 and 544 cells, respectively, were captured on each coverslip from the three experiments and quantitatively evaluated using ImageJ software. Examples of fluorescence images are shown.

(B) The number of nuclei recognized with DAPI staining in α-SMA-positive (α-SMA^{+}) areas was counted as α-SMA^{+} cells (DAPI^{+} α-SMA^{+} cells). The number of LEF1-positive nuclei in α-SMA^{+} areas was counted as LEF1 and α-SMA double-positive (LEF1^{+} α-SMA^{+}) cells. The percentage of LEF1^{+} α-SMA^{+} cells in the total α-SMA^{+} cells is shown as a dot plot. Each dot represents the quantitation result from captured images. Means (thick horizontal lines) and SD (error bars) are indicated.

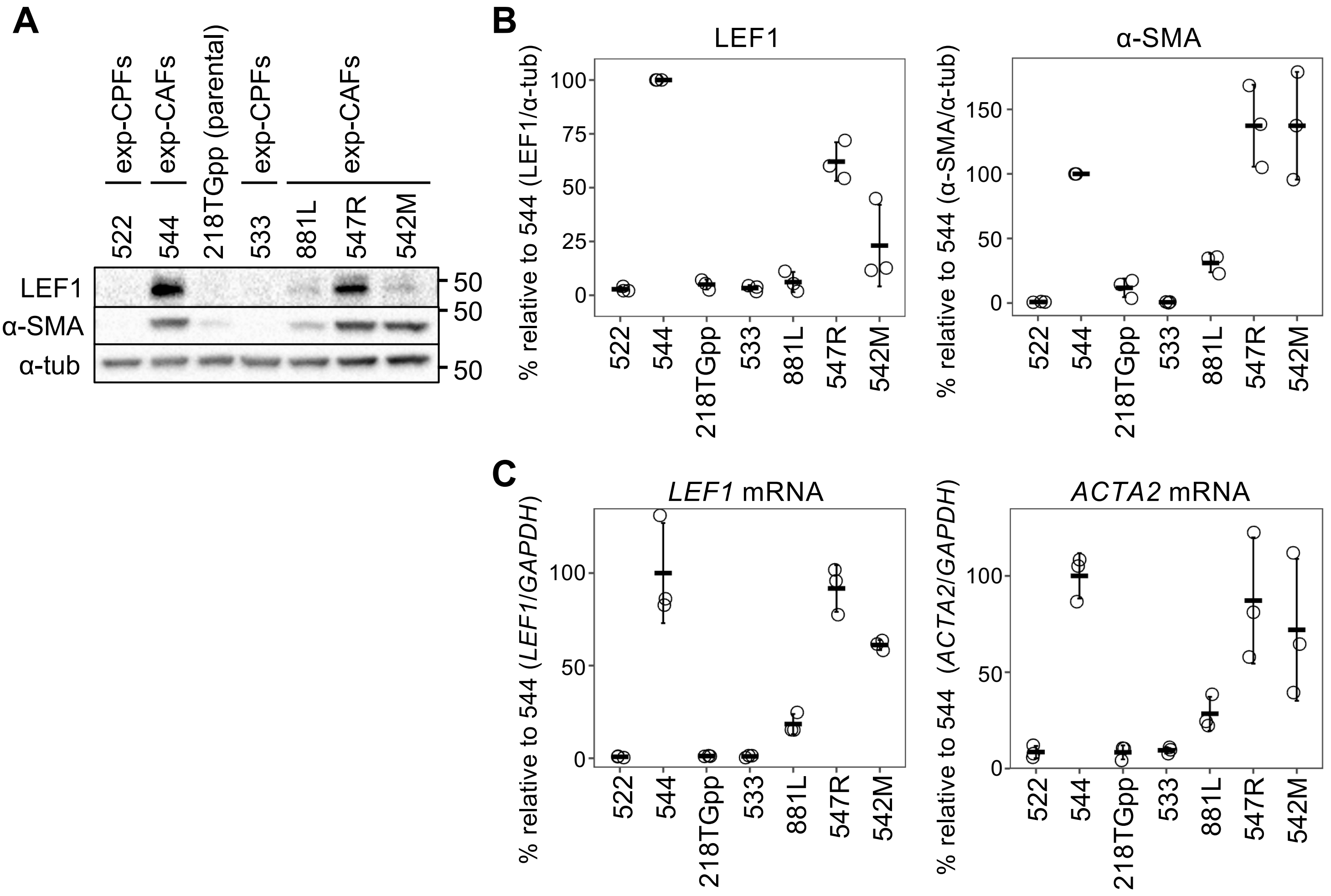

Figure S3

Figure S3. Analysis of LEF1 expression in experimentally generated fibroblasts

(A) Western blot analysis to assess LEF1 and α-SMA expression, with α-tubulin (α-tub) as a loading control. In addition to 544 and 522 cells, a few more fibroblast lines were examined. 218TGpp cells are the parental fibroblasts used to generate exp-CAFs and exp-CPFs (2). 544, 881L, 547R and 542M cell lines were generated through incubation with breast cancer cells *in vivo* for 242, 85, 170 and 242 days, respectively. 522 and 533 cells were similarly incubated *in vivo* without breast cancer cells for 242 days. Results of 522 and 544 cells are also shown in the main Figure 1A.

(B) Quantitative analysis of western blot band intensities. The band intensities of LEF1 and α-SMA were normalized with that of α-tubulin. Then, normalized intensities of LEF1/α-tub and α-SMA/α-tub from 544 cells were set as 100, and those from other cell lines were calculated relative to them at each experiment set. Results from three independent experiments are shown as dot plots with the means (thick horizontal lines) with SD (error bars).

(C) RT-qPCR analysis to measure *LEF1* and *ACTA2* mRNA expression levels. Their expression levels were normalized against those of *GAPDH*. The normalized levels of *LEF1*/*GAPDH* and *ACTA2*/*GAPDH* of 544 cells from three independent experiments were averaged and expressed as 100 and those of all samples were calculated relative to them. Results from the three independent experiments are shown as dot plots with the means (thick horizontal lines) with SD (error bars).

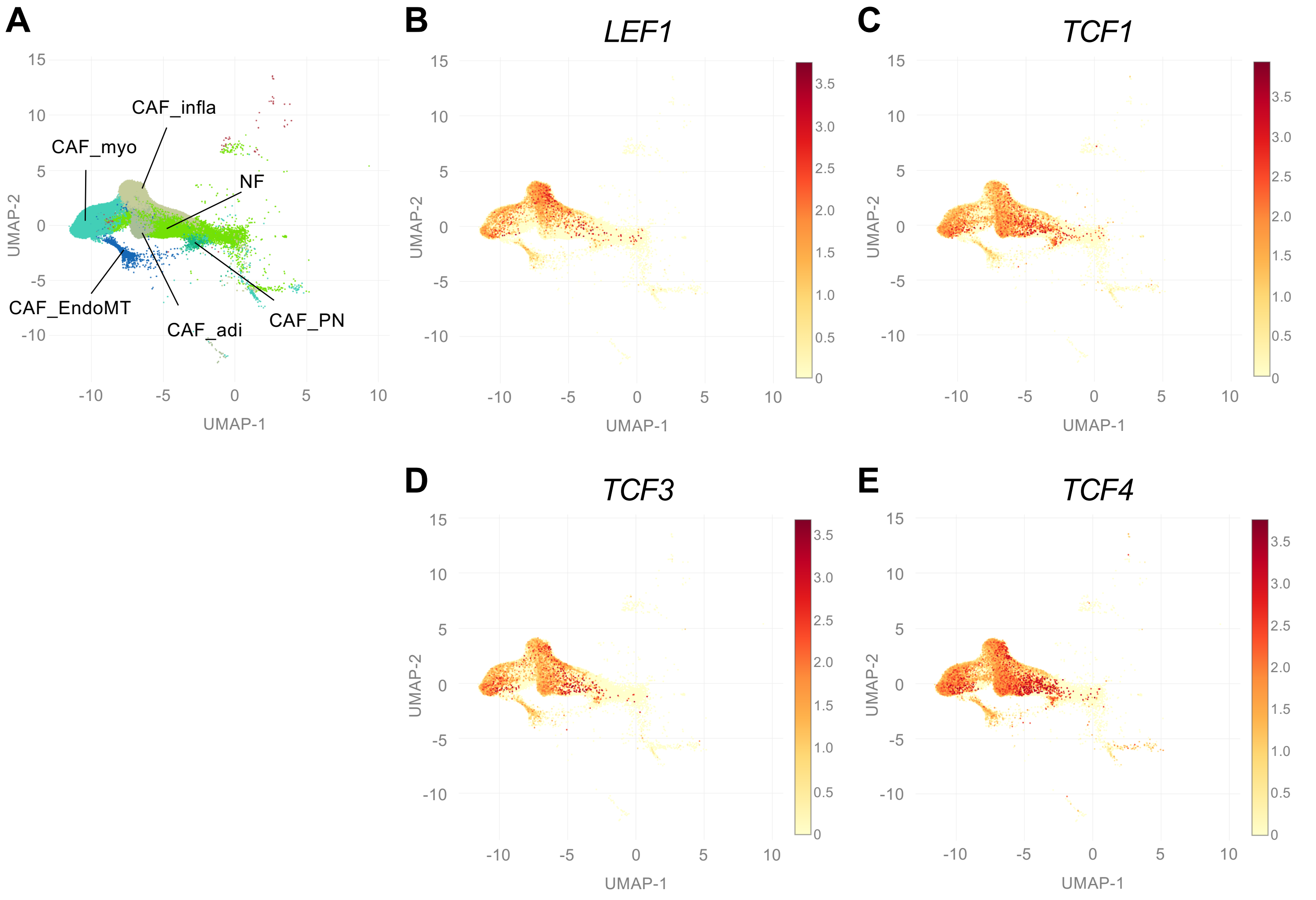

Figure S4

Figure S4. Distribution of TCF/LEF1 family gene expression in fibroblasts from various cancer types

(A) Single-cell RNA-seq data in Luo et al (3) were reanalyzed and presented as UMAP plots using their browser interface. Fibroblasts were extracted and divided into cancer-associated myofibroblasts (CAF_myo), inflammatory CAF (CAF_infla), CAFs exhibiting endothelial-mesenchymal transition (CAF_EndoMT), adipogenic CAF(CAF_adi), fibroblast-like peripheral nerve cells (CAF_PN), and normal fibroblasts (NF).

(B-E) UMAP plots of the TCF/LEF1 family genes, *LEF1* (B), *TCF1* (C), *TCF3* (D), and *TCF4* (E).

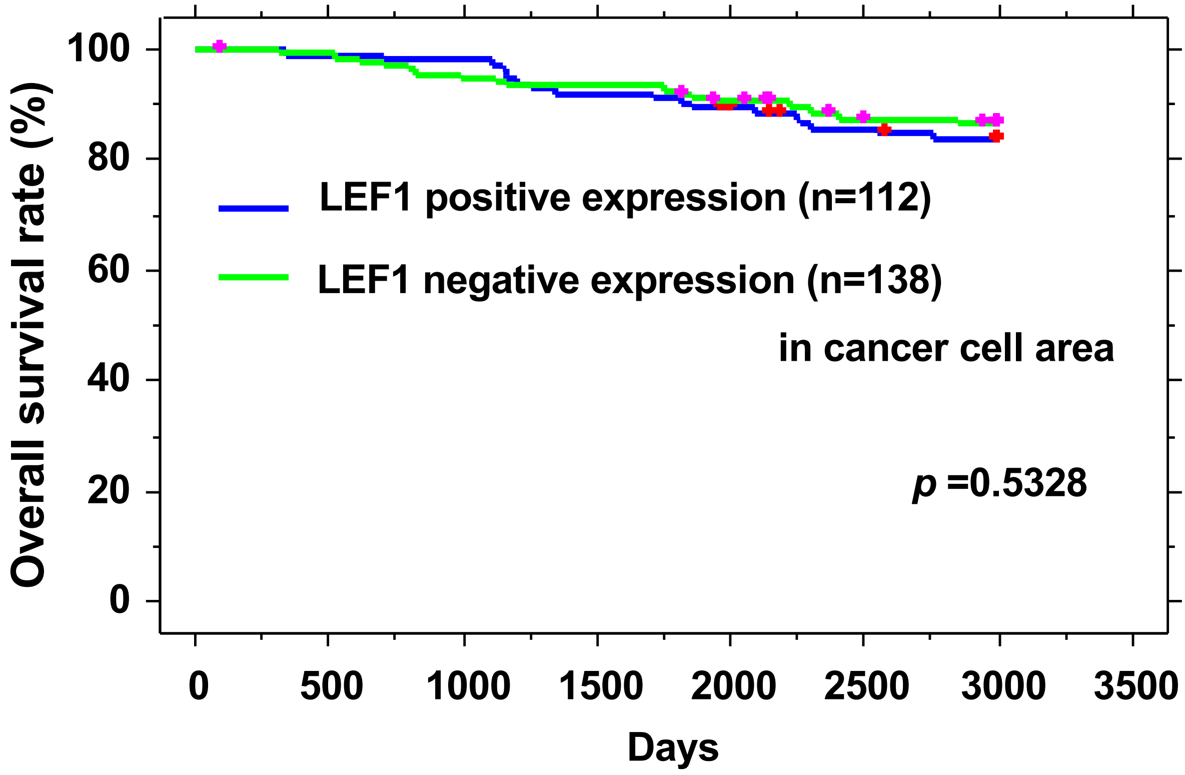

Figure S5

Figure S5. Kaplan–Meier survival plot of 250 human breast cancer patients based on LEF1 staining in cancer cell area of their tumors

When ≥10% of the cancer cell region was positive for LEF1 staining, specimens were considered positive (p = 0.5328 by log-rank test).

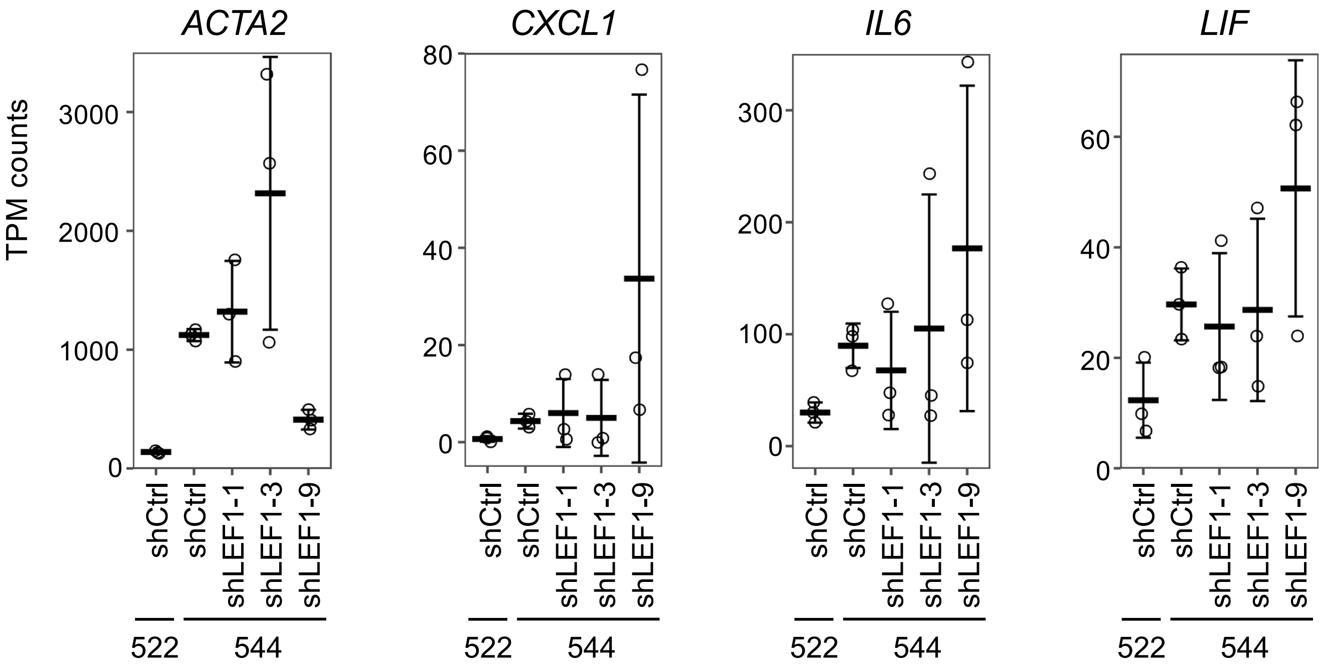

gene	log2FC	padj
ACTA2	0.204	0.925
CXCL1	1.694	0.580

gene	log2FC	padj
IL6	0.261	0.909
LIF	0.050	0.967

Figure S6

Figure S6. Levels of CAF markers in exp-CAF 544 cells and exp-CPF 522 cells expressing the indicated shRNA

TPM counts from RNA-seq of the indicated mRNAs in 522-shCtrl, 544-shCtrl, 544-shLEF1-1, 544-shLEF1-3, and 544-shLEF1-9 from three independent experiments are shown as dot plots with the means (thick horizontal lines) and SD (error bars). The table shows values of Log_2 FC and padj of the indicated genes obtained with DESeq2 analysis using RNA-seq results.

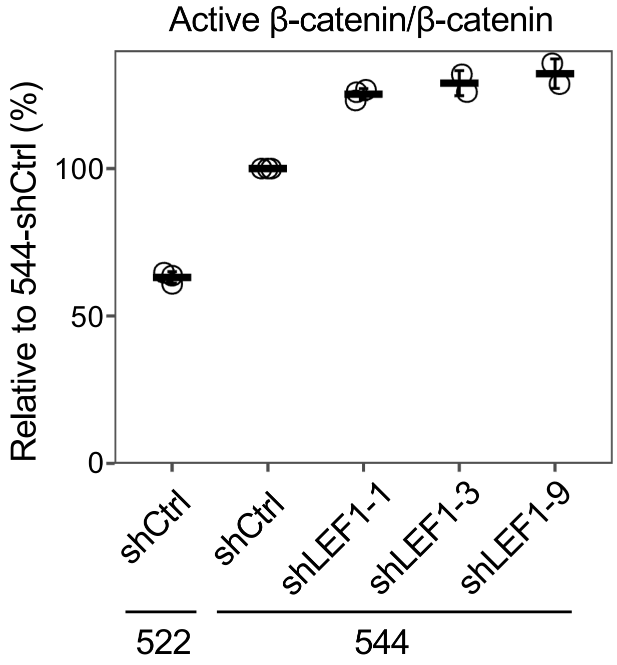

Figure S7

Figure S7. LEF1 knockdown does not downregulate the levels of active β-catenin in exp-CAF 544 cells

Quantitative analysis of western blot results for active β-catenin (unphosphorylated form) and β-catenin presented in the main Figure 6D. The band intensities of active form β-catenin were normalized with those of total β-catenin. Then, normalized intensity of active form β-catenin/total β-catenin from 544-shCtrl was expressed as 100, and those from other cells were calculated relative to this at each experiment set. Results from three independent experiments are shown as dot plots with the means (thick horizontal lines) with SD (error bars).

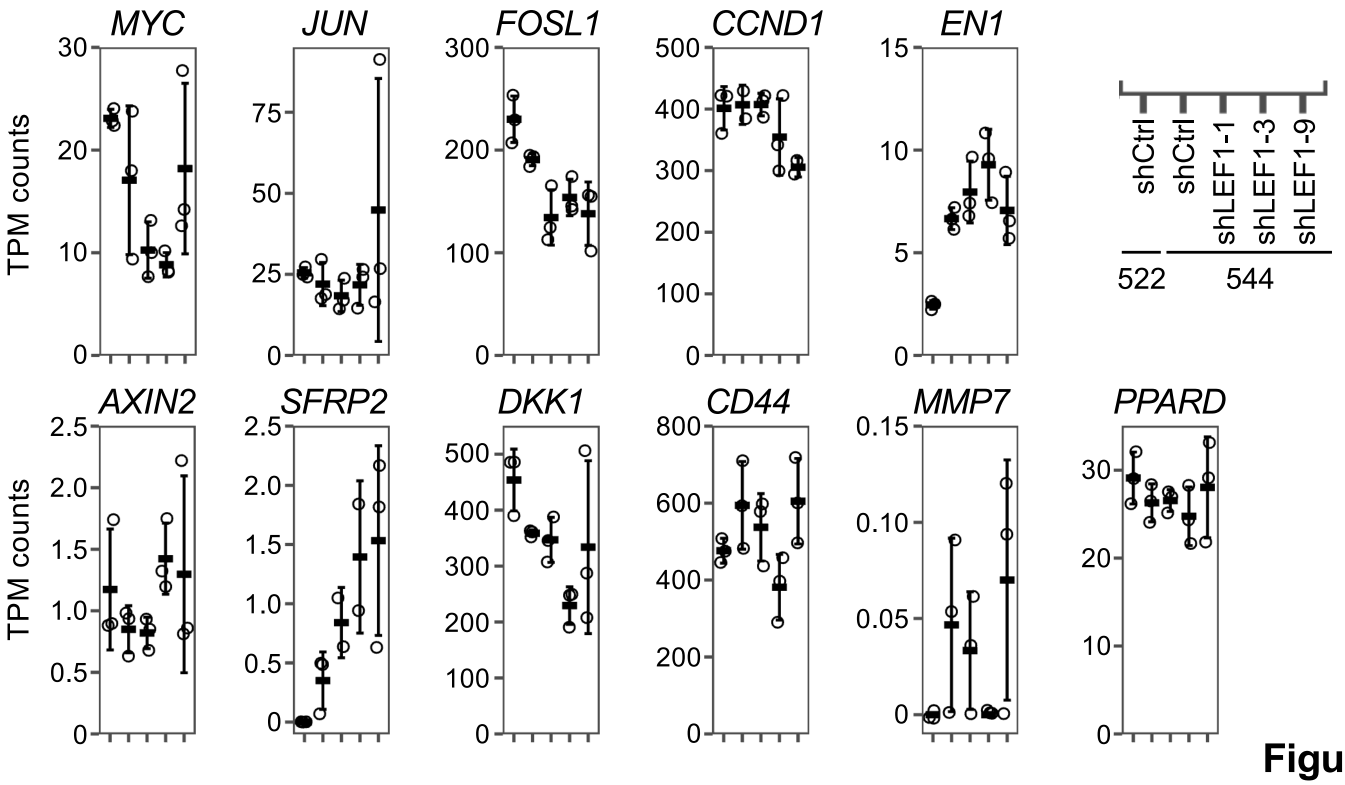

Figure S8

Figure S8. Levels of Wnt/β-catenin pathway target genes in exp-CAF 544 cells and exp-CPF 522 cells expressing the indicated shRNA

TPM counts from RNA-seq of the indicated mRNAs in 522-shCtrl, 544-shCtrl, 544-shLEF1-1, 544-shLEF1-3, and 544-shLEF1-9 from three independent experiments are shown as dot plots with the means (thick horizontal lines) and SD (error bars). Assignment of lanes in the graphs is indicated above the *PPARD* graph.

NBBP

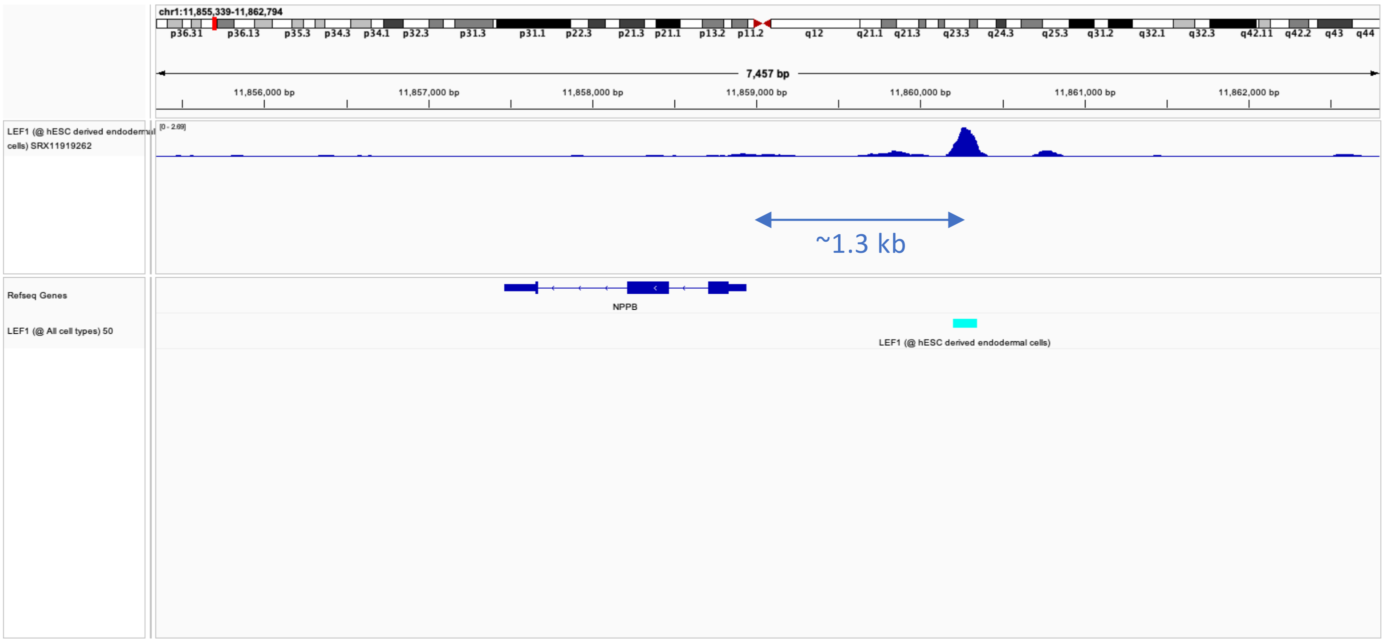

DYNC1LI2-DT

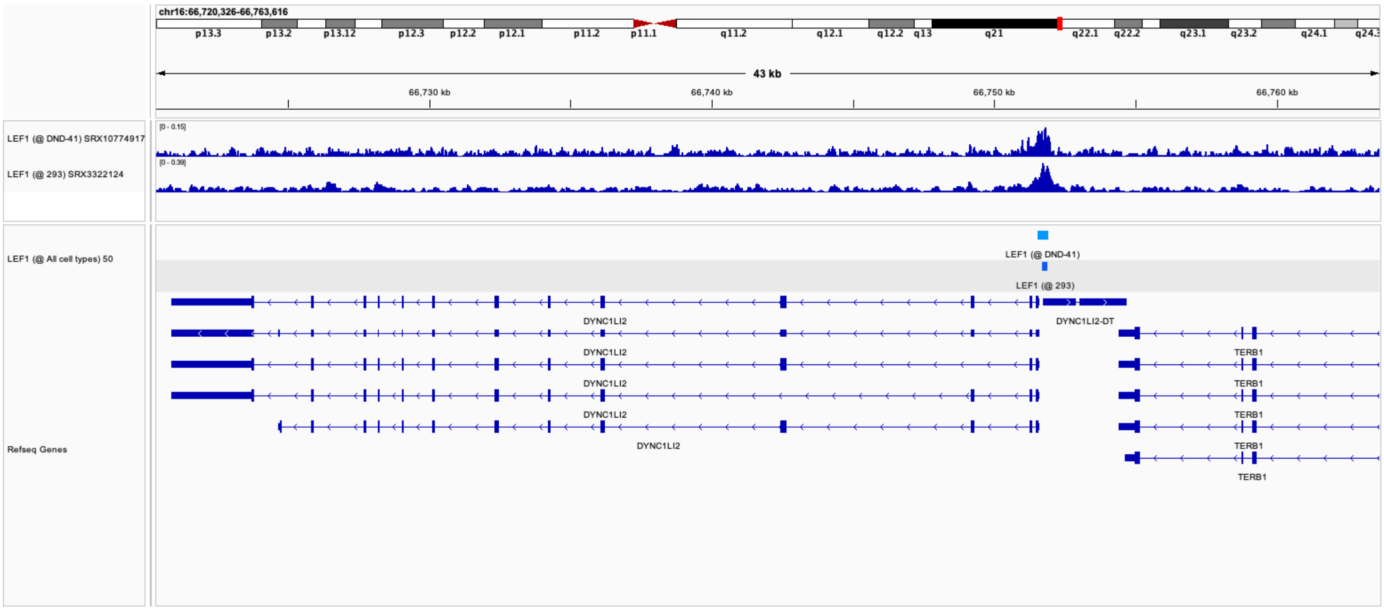

CMKLR2

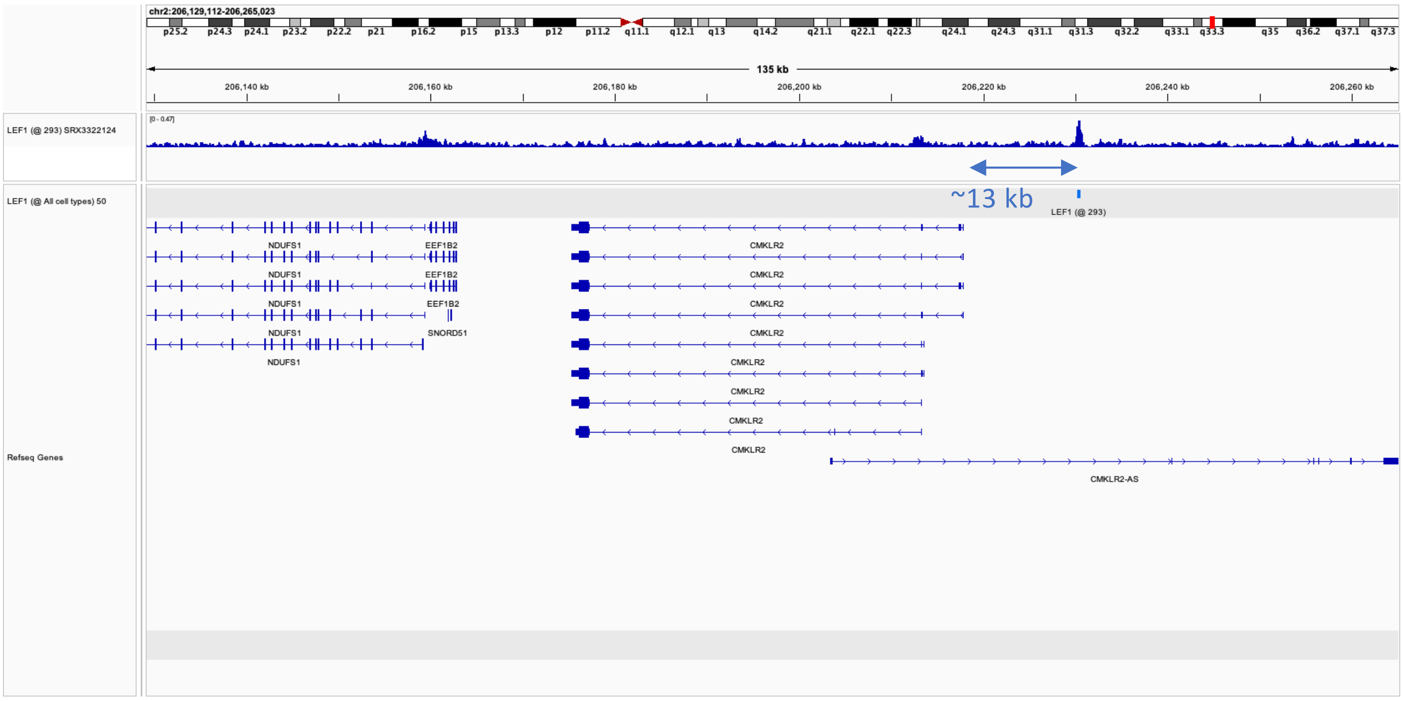

Figure S9

CCDC190

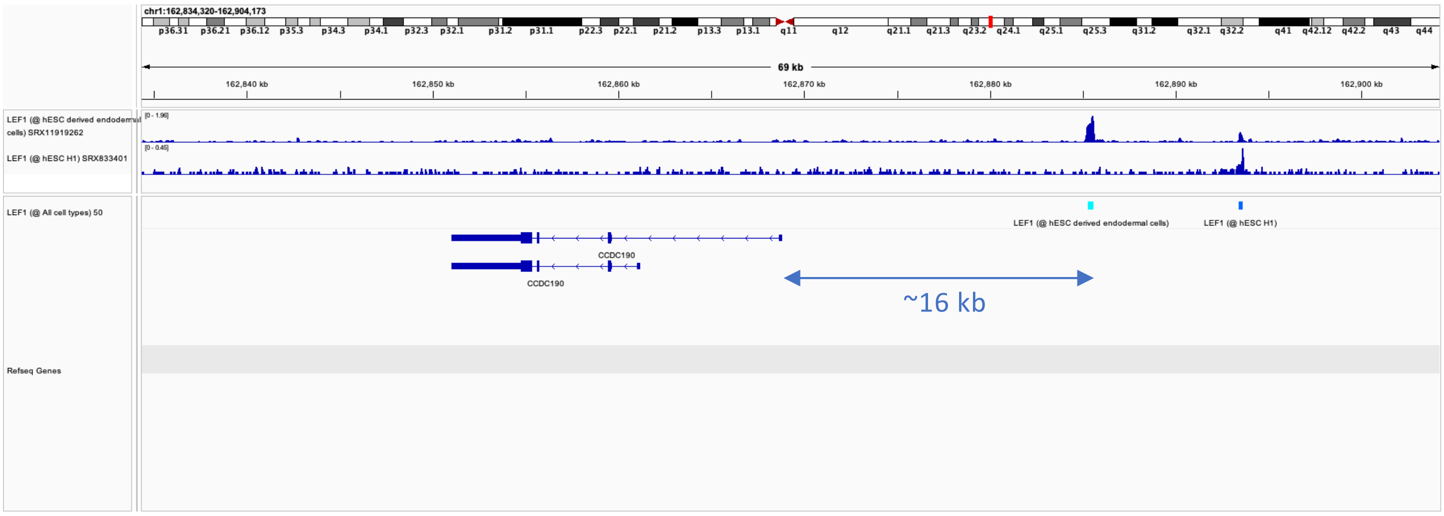

GATA6

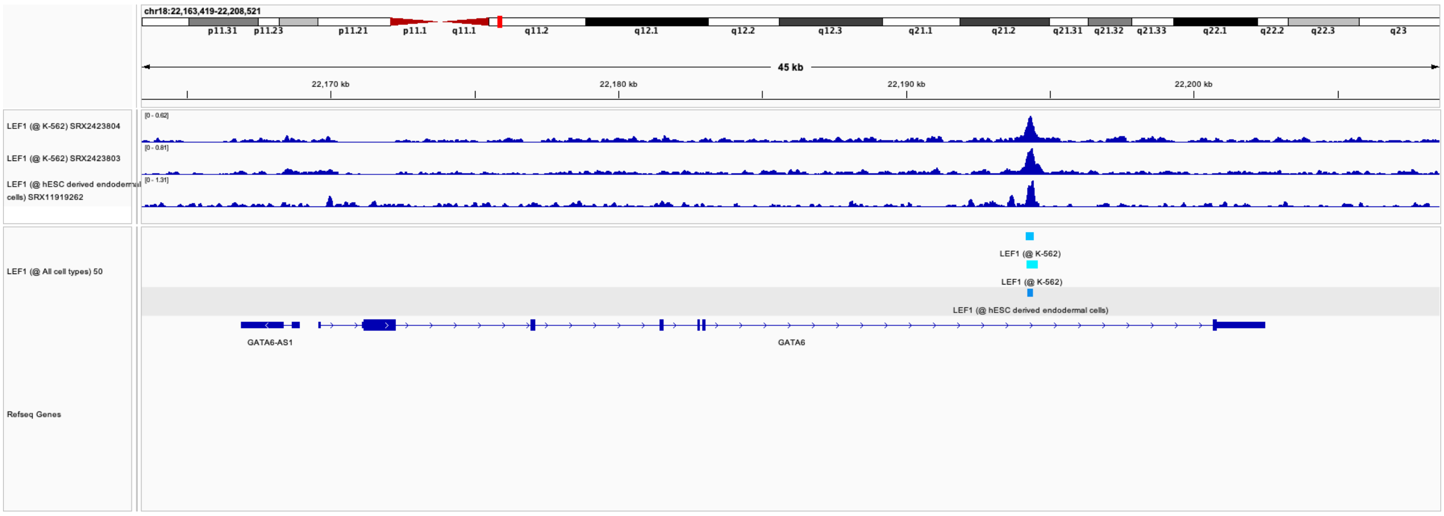

Figure S9, continued

Figure S9. LEF1 binding in published ChIP-seq data

Binding of LEF1 to and around the 5′-ends of the 13 genes in the main Figure 6E was analyzed in published human LEF1- ChIP-seq data using ChIP-Atlas (4). Image was generated using ChIP-Atlas: Peak Browser and shown in Integrative Genomics Viewer (5). LEF1 binding sites appear to be associated with 5 genes, NBBP, DYNC1LI2-DT, CMKLR2, CCDC190, and GATA6, out of the 13 genes.

References for Supplementary Data

(1) Koyama, Y., Okazaki, H., Shi, Y., Mezawa, Y., Wang, Z., Sakimoto, M., Ishizuka, A., Ito, Y., Koyama, T., Daigo, Y., Takano, A., Miyagi, Y., Yokose, T., Yamashita, T., Sugahara, K., Hino, O., Yang, L., Maruyama, R., Katakura, A., Yasukawa, T. and Orimo, A. (2023) Increased RUNX3 expression mediates tumor-promoting ability of human breast cancer-associated fibroblasts. *Cancer Med*, **12**, 18062-18077.

(2) Kojima Y, Acar A, Eaton EN, et al. Autocrine TGF-beta and stromal cell-derived factor-1 (SDF-1) signaling drives the evolution of tumor-promoting mammary stromal myofibroblasts. *Proc Natl Acad Sci U S A*. 2010; 107: 20009-20014.

(3) Luo H, Xia X, Huang LB, et al. Pan-cancer single-cell analysis reveals the heterogeneity and plasticity of cancer-associated fibroblasts in the tumor microenvironment. *Nat Commun*. 2022; 13: 6619.

(4) Zou Z, Ohta T, Miura F, Oki S. ChIP-atlas 2021 update: a data- mining suite for exploring epigenomic landscapes by fully in- tegrating ChIP-seq, ATAC-seq and bisulfite-seq data. *Nucleic Acids Res*. 2022;50(W1):W175-W182.

(5) Robinson JT, Thorvaldsdóttir H, Winckler W, et al. Integrative Genomics Viewer. Nat. Biotechnol. 2011; 29: 24-26.
